# Changes in community composition and functional diversity of European bats under climate change

**DOI:** 10.1111/cobi.70025

**Published:** 2025-04-01

**Authors:** Penelope C. Fialas, Luca Santini, Danilo Russo, Francisco Amorim, Hugo Rebelo, Roberto Novella‐Fernandez, Francisco Marques, Adi Domer, Adriana Vella, Adriano Martinoli, Aleksandra Figurek, Asaf Tsoar, Attila Sandor, Carlos Ibanez, Carmi Korine, Christian Kerbiriou, Christian Voigt, Claire Mifsud, Csaba Jére, Dalhoumi Ridha, Damiano Preatoni, Daniela Hamidović, Eeva‐Maria Tidenberg, Emrah Çoraman, Fiona Mathews, Fulgencio Lison, Furmankiewicz Joanna, Gunars Petersons, Hiba Loumassine, Inazio Garin, István Csősz, Jaan Liira, Javier Juste, Jean François Julien, Jeroen van der Kooij, Josić Darija, Joxerra Aihartza, Katrine Eldegard, Kendra Phelps, Kevin J. Olival, Kipson Marina, Leonardo Ancillotto, Lesiński Grzegorz, Levente Barti, Lisette Cantú Salazar, Luciano Bosso, Luisa Rodrigues, Luke Hamel, Marcel Uhrin, Maria Mas, Natasa Cerekovic, Nia Toshkova, Niamh Roche, Oliver Kalda, Ostaizka Aizpurua, Panagiotis Georgiakakis, Peter Kanuch, Primož Presetnik, Rasit Bilgin, Reed April McKay, Rnjak Dina, Rnjak Goran, Ruczyński Ireneusz, Rune Sørås, Solène Robert, Stéphane Aulagnier, Stephanie Kramer‐Schadt, Suren Gazaryan, Szilárd‐Lehel Bücs, Tarkan Yorulmaz, Torsten Stjernberg, Ulla‐Maija Liukko, Victoria Nistreanu, Viesturs Vintulis, Viktoriia Radchuk, Xavier Puig‐Montserrat, Yves Bas, Maja Zagmajster, Marcin Zegarek, Zrnčić Vida, Orly Razgour

**Affiliations:** ^1^ Department of Biosciences University of Exeter Exeter UK; ^2^ Department of Biology and Biotechnologies “Charles Darwin” Sapienza University of Rome Rome Italy; ^3^ Laboratory of Animal Ecology and Evolution (AnEcoEvo), Dipartimento di Agraria Università degli Studi di Napoli Federico II Portici Italy; ^4^ CIBIO, Centro de Investigação em Biodiversidade e Recursos Genéticos, InBIO Laboratório Associado Universidade do Porto Vairão Portugal; ^5^ Department of Global Change Ecology, Universitat Wurzburg Wurzburg Germany; ^6^ Centre for Ecology, Evolution and Environmental Changes, Faculty of Sciences University of Lisbon Lisbon Portugal; ^7^ Department of Life Sciences Ben‐Gurion University of the Negev Beer Sheva Israel; ^8^ Conservation Biology Research Group, Department of Biology University of Malta Msida Malta; ^9^ Unità di Analisi e Gestione delle Risorse Ambientali ‐ Giuso Tosi Research Group, Dipartimento di Scienze Teoriche e Applicate Università degli Studi dell'insubria Varese Italy; ^10^ Faculty of Agriculture, Bosnia and Herzegovina University of Banja Luka Banja Luka Bosnia and Herzegovina; ^11^ Mitrani Department of Desert Ecology, Jacob Blaustein Institutes for Desert Research Ben‐Gurion University of the Negev Midreshet Ben‐Gurion Israel; ^12^ Department of Parasitology and Parasitic Diseases, Faculty of Veterinary Medicine University of Agricultural Sciences and Veterinary Medicine Cluj‐Napocac Cluj‐Napoca Romania; ^13^ Department of Ecology and Evolution Estacion Biologica Doñana (CSIC) Sevilla Spain; ^14^ Centre d'´Ecologie et des Sciences de la Conservation (CESCO), Muséum national d'Histoire naturelle, Centre National de la Recherche Scientifique Sorbonne Université Paris France; ^15^ Station de Biologie Marine Concarneau France; ^16^ Leibniz Institute for Zoo and Wildlife Research Berlin Germany; ^17^ Conservation Biology Research Group, Department of Biology, University of Malta Msida Malta; ^18^ Myotis Bat Conservation Group Miercurea Ciuc Romania; ^19^ Laboratory of Environmental Biomonitoring, Bizerte Faculty of Science University of Carthage Zarzouna Tunisia; ^20^ Institute for Environment and Nature Ministry of Economy and Sustainable Development Zagreb Croatia; ^21^ Finnish Museum of Natural History (LUOMUS) University of Helsinki Helsinki Finland; ^22^ Eurasia Institute of Earth Sciences, Department of Ecology and Evolution Istanbul Technical University Maslak Turkey; ^23^ School of Life Sciences University of Sussex Brighton UK; ^24^ Wildlife Ecology and Conservation Lab, Departamento de Zoología, Facultad de Ciencias Naturales y Oceanográficas Universidad de Concepción Concepción Chile; ^25^ Faculty of Behavioural Ecology University of Wroclaw Wroclaw Poland; ^26^ Faculty of Veterinary Medicine Latvia University of Life Sciences and Technologies Jelgava Latvia; ^27^ Naturalia Environnement Avignon France; ^28^ Department of Zoology and Animal Cell Biology, Facultyof Science and Technology, University of the BasqueCountry UPV/EHU, Sarriena Z.G., The Basque Country Leioa Spain; ^29^ Institute of Ecology and Earth Sciences University of Tartu Tartu Estonia; ^30^ Nature Education Research and Consultancy van der Kooij Slattum Norway; ^31^ Museum für Naturkunde, Leibniz Institute for Evolution and Biodiversity Science Berlin Germany; ^32^ Environmental Sciences and Natural Resource Management Norwegian University of Life Sciences Ås Norway; ^33^ EcoHealth Alliance New York New York USA; ^34^ Department of Zoology, Faculty of Science Charles University in Prague Prague Czech Republic; ^35^ Institute for the Research on Terrestrial Ecosystems (IRET) National Research Council (CNR) Sesto Fiorentino Italy; ^36^ Institute of Animal Science Warsaw University of Life Sciences (SGGW) Warsaw Poland; ^37^ Luxembourg Institute of Science and Technology Esch‐sur‐Alzette Luxembourg; ^38^ Institute for Agriculture and Forestry Systems in the Mediterranean National Research Council of Italy Portici Italy; ^39^ Instituto da Conservação da Natureza e das Florestas Lisbon Portugal; ^40^ Department of Zoology, Institute of Biology and Ecology University of Pavol Jozef Šafárik Košice Slovakia; ^41^ BiBio Research Group Natural Sciences Museum of Granollers Granollers Spain; ^42^ National Museum of Natural History Bulgarian Academy of Sciences Sofia Bulgaria; ^43^ Bat Conservation Ireland Dublin Ireland; ^44^ Center for Evolutionary Hologenomics, GLOBE Institute University of Copenhagen Copenhagen Denmark; ^45^ Natural History Museum of Crete University of Crete Heraklion Greece; ^46^ Institute of Forest Ecology Slovak Academy of Sciences Zvolen Slovakia; ^47^ Centre for Cartography of Fauna and Flora Ljubljana Office Ljubljana Slovenia; ^48^ Geonatura Ltd. Zagreb Croatia; ^49^ Mammal Research Institute PAS Białowieża Poland; ^50^ Department of Biology Norwegian University of Science and Technology Trondheim Norway; ^51^ PatriNat (Office Français de la Biodiversité (OFB) – Centre National de la Recherche Scientifique (CNRS) – Muséum National d'Histoire Naturelle (MNHN)) Paris France; ^52^ Comportement et Ecologie de la Faune sauvage Auzeville‐Tolosane France; ^53^ UNEP/EUROBATS Secretariat Bonn Germany; ^54^ Centre for Bat Research and Conservation Cluj‐Napoca Romania; ^55^ Program of Hunting and Wildlife, Department of Forestry, Food and Agriculture Vocational School University of Çankırı Karatekin Çankırı Turkey; ^56^ Finnish Environment Institute Helsinki Finland; ^57^ Institute of Zoology Moldova State University Chisinau Moldova; ^58^ Institute of Biology, University of Latvia Salaspils Latvia; ^59^ University of Ljubljana, Subterranean Biology Lab (SubBioLab) Department of Biology, Biotechnical Faculty, Kongresni Ljubljana Slovenia

**Keywords:** bats, community composition, functional composition, global change, species distribution modeling, Cambio global, composición de la comunidad, composición funcional, murciélagos, modelización de la distribución de especies

## Abstract

Climate change is predicted to drive geographical range shifts that will result in changes in species diversity and functional composition and have potential repercussions for ecosystem functioning. However, the effect of these changes on species composition and functional diversity (FD) remains unclear, especially for mammals, specifically bats. We used species distribution models and a comprehensive ecological and morphometrical trait database to estimate how projected future climate and land‐use changes could influence the distribution, composition, and FD of the European bat community. Future bat assemblages were predicted to undergo substantial shifts in geographic range and trait structure. Range suitability decreased substantially in southern Europe and increased in northern latitudes. Our findings highlight the potential for climate change to drive shifts in bat FD, which has implications for ecosystem function and resilience at a continental scale. It is important to incorporate FD in conservation strategies. These efforts should target species with key functional traits predicted to be lost and areas expected to experience losses in FD. Conservation strategies should include habitat and roost protection, enhancing landscape connectivity, and international monitoring to preserve bat populations and their ecosystem services.

## Abstract (es)

Cambios en la composición de la comunidad y la diversidad funcional de murciélagos europeos bajo el cambio climático

**Resumen:**

Se prevé que el cambio climático provocará desplazamientos geográficos que alterarán la diversidad de las especies y su composición funcional, con posibles repercusiones en el funcionamiento de los ecosistemas. Sin embargo, el efecto de estos cambios sobre la composición de las especies y la diversidad funcional aun no es claro, especialmente en el caso de los mamíferos, en concreto los murciélagos. Utilizamos modelos de distribución de especies y una base de datos integral de rasgos ecológicos y morfométricos para estimar cómo los futuros cambios previstos en el clima y el uso del suelo podrían influir en la distribución, composición y diversidad funcional de la comunidad europea de murciélagos. Se predijo que los futuros conjuntos de murciélagos sufrirían cambios sustanciales en su distribución geográfica y en la estructura de sus atributos. La idoneidad del área de distribución disminuyó sustancialmente en el sur de Europa y aumentó en las latitudes septentrionales. Nuestros resultados ponen de relieve la posibilidad de que el cambio climático provoque cambios en la diversidad funcional de los murciélagos, lo que tiene implicaciones para la función y la resiliencia de los ecosistemas a escala continental. Es importante incorporar la diversidad funcional a las estrategias de conservación. Estos esfuerzos deberían centrarse en las especies con rasgos funcionales clave que se prevé que se pierdan y en las zonas en las que se espera que se produzcan pérdidas de diversidad funcional. Las estrategias de conservación deberían incluir la protección del hábitat y de los dormideros, la mejora de la conectividad del paisaje y el seguimiento internacional para preservar las poblaciones de murciélagos y sus servicios ecosistémicos.

## INTRODUCTION

Climate change is a key and growing threat to biodiversity (IPCC, 2022) that is driving substantial shifts in patterns of species distribution and diversity worldwide (Chen et al., 2011; Parmesan & Yohe, 2003; Walther et al., 2002). Under future climate change scenarios, the geographic range for many species is predicted to expand or contract in size or shift along latitudinal and elevational gradients (Dullinger et al., 2012; Rebelo et al., 2010; Thomas et al., 2004). The variation in species‐specific responses to climate change can lead to the emergence of new species assemblages differing in compositions and functions from those observed today (Williams & Jackson, 2007). Such changes can influence biotic interactions (Blois et al., 2013), alter ecosystem processes (Loreau et al., 2001), and possibly trigger trophic cascades (Mäntylä et al., 2011). The impacts of climate change on species distributions will be exacerbated by ongoing anthropogenic land‐use change in the form of habitat loss and fragmentation, which is already responsible for an extensive decline in wildlife abundance and functional diversity (FD) across the globe (Brodie et al., 2021; Maxwell et al., 2016). Such land‐use changes are also likely to impair species’ ability to shift their ranges in response to future climate change (Robillard et al., 2015).

Species distributional changes have consequences at the community level. Competition may emerge when newly arriving species use the same limited resources that local species rely on (e.g., Tannerfeldt et al., 2002). If generalist species colonize areas that have become climatically suitable for them, they may displace local, more habitat specialists, as seen in small mammal assemblages at high elevations (Clavel et al., 2011; Rowe et al., 2010). Thus, climate‐change‐induced distributional shifts can lead to a simplification of communities as opportunistic species expand to new areas, and specialist species may experience range contractions and ultimately disappear (McKinney & Lockwood, 1999). These changes could have further repercussions on ecosystem functions due to shifts in functional composition (Thuiller et al., 2006). Replacing local species with functionally different species (Rosenfeld, 2002) can have substantial and potentially unpredictable impacts on community structure and ecosystem functioning. Furthermore, climate‐change‐induced range shifts in mammals can lead to an increase in cross‐species transmission of pathogens, which can affect population viability (Carlson et al., 2022). Hence, understanding and assessing such changes in range, assemblage structure, and FD is an important step toward developing effective conservation strategies, particularly in regions where ecosystem functions are likely to be affected by climate change (McCarthy et al., 2011; Roy et al., 2015).

Bats offer an important case study for community‐level responses to climate change due to their high diversity, distinct ecological roles, and sensitivity to environmental changes. Bats, accounting for 20% of mammalian species, are widely distributed and play key ecological roles, such as insect suppression (Jones et al., 2009; Kunz et al., 2011). Bats are thought to be particularly sensitive to climate change, especially to temperature increases and extreme events, because their high body surface‐to‐volume ratios due to large wing and tail membranes mean they are prone to dehydration (Korine et al., 2016). Changing temperatures can affect various aspects of bat physiology and ecology, from torpor duration and intensity to reproduction requirements and food availability, which can lead to demographic and spatial responses to climate change (reviewed in Festa et al. [2022] and Sherwin et al. [2013]). Conversely, due to their flight ability, some bat species have a greater capacity for long‐distance dispersal (Hutterer et al., 2005) relative to small terrestrial mammals (e.g., rodents and shrews) and consequently higher range shift potential in the face of climate change, assuming the availability of habitats.

Numerous studies have demonstrated the impact of climate change on the distribution, activity, survival, and reproductive success of bats. Yet, impacts at the community and functional level have not been explored (Festa et al., 2022). To address this knowledge gap, we predicted how projected future climate and land‐use changes may influence the distribution, composition, and FD of the European bat community. To this end, we collected a unique data set of more than 14,000 observations through a cross‐European collaborative network and employed ensemble forecasting to predict the future range suitability for 37 European bat species. We integrated these predictions with species traits to estimate future trajectories in community composition and FD. We hypothesized that climatic suitability increases in northern Europe and decreases in southern Europe, resulting in a shift in patterns of species richness, and that shifts in climatic suitability across Europe lead to changes in community composition and FD in northern and southern latitudes. We predicted that gains in species richness in northern Europe will result in more pronounced changes in community composition and FD than range losses in southern Europe because there are fewer species in northern Europe.

## METHODS

### Study area

The study area encompassed Europe and the North African and Middle Eastern regions bordering the Mediterranean Sea (Appendix S1). We extended the study area to North Africa and the Middle East to include the southern range limits of the distribution of some European bat species (e.g., *Pipistrellus kuhlii*, *Rhinolophus mehelyi*, *Barbastella barbastellus*) and hence capture their full range of climatic tolerances. This area covered the geographic range of the most common European species and the European near‐endemic bat species (Hackländer & Zachos, 2020). We included only European bat species because of a lack of information on non‐European species for assessing changes in FD.

### Bat species occurrence data

We collected a comprehensive data set of unpublished location records from bat experts from across Europe through the ClimBats COST Action network (CA18107). We obtained data from 66 data providers for 29 countries, including location records from 1980. We supplemented our data set with location records extracted from the literature for North Africa (Appendix S2) and the EUROBATS data set (www.eurobats.org). We did not use GBIF records because of concerns about their accuracy with cryptic species identification and their associated spatial bias (Beck et al., 2014). This approach ensured our data set was robust and reliable and avoided potential pitfalls associated with commonly used databases.

We removed duplicate records and thinned the records of each species to 40 km with the R package spThin (Aiello‐Lammens et al., 2015) to reduce spatial clustering. This value resulted in the best balance between retaining a sufficient number of records and reducing clustering in more densely sampled countries. The final data set included 14,638 location records of 37 bat species. Species presence data ranged between 62 locations for *Plecotus kolombatovici* and 929 for *Pipistrellus pipistrellus* (average 393.05 location records [SD 234.58]) (Appendix S3). These data are available from Dryad https://doi.org/10.5061/dryad.2z34tmpx6.

### Environmental variables

We selected 8 environmental variables that are ecologically meaningful for bats and likely to limit their distribution, based on knowledge of the ecology of European bats (Table 1). We combined climatic, land cover, and topographic variables. We initially included 6 bioclimatic variables downloaded from CHELSA‐Climate (Karger et al., 2017) at a 30‐arc‐second resolution (∼1 km), 2 land cover variables, proportion of forested and urban area derived from the 300‐m‐resolution Globio4 map (Schipper et al., 2020), and one topographic variable, which was calculated from the Copernicus Digital Elevation Model data set at a 30‐m resolution (topographic ruggedness index) (Appendix S4). We used the R package raster (Hijmans et al., 2015) to upscale variables to approximately 5 km, corresponding to the average home range of bat species (Stebbings & Griffith, 1986), and thus include the area likely used by the bat around each location record. We removed intercorrelated variables with variation inflation factor >10 and Spearman correlation rho >|0.7|. The final data set included 5 bioclimatic variables, 2 land cover variables, and one topographic variable (Table 1).

**TABLE 1:** Environmental variables used for European bat species distribution modeling.

Variable abbreviation	Description
CHELSA Bio04	Temperature seasonality
CHELSA Bio05	Maximum temperature of warmest month
CHELSA Bio12	Annual precipitation
CHELSA Bio15	Precipitation seasonality
CHELSA bio18	Precipitation of warmest quarter
Globio forested	Land cover—forest
Globio urban	Land cover—urban
DEM ruggedness	Topographic ruggedness index

To forecast species distribution under future scenarios, we used projected climatic data for 2041–2060 based on the RCP 4.5 (representative concentration pathway) and RCP 8.5 scenarios (medium‐ and high‐emission scenarios) (IPCC, 2022) and 3 general circulation models (HadGEM2, IPSL‐CM5A, and MPI‐ESM) (www.chelsa‐climate.org) chosen because of their European focus (IPCC, 2013). Future land cover projections were based on the Globio4 model and the SSP3 and SSP5 scenarios (Schipper et al., 2020). We focused on the results of the high‐emission scenarios (RCP 8.5 and SSP5) to highlight the consequences of inaction on climate change.

### Modeling procedure

We generated species distribution models (SDMs) to predict the impact of climate change on the ranges of 37 bat species across the study area. For each species, occurrence records were split into 70% training and 30% testing data. For each species range, we generated 10,000 random background points with the dismo package (Hijmans et al., 2017). Following best practices (Araújo et al., 2019), SDMs were fitted using an ensemble approach with 4 different algorithms: generalized additive models (McCullagh & Nelder, 1989), boosted regression trees (Elith et al., 2008), artificial neural networks (Lek & Guégan, 1999), and maximum entropy (Phillips et al., 2006). We used the R package biomod2 4.1.2 (Thuiller et al., 2016) to generate the models. We adjusted the model parameters of each algorithm for each species with SDMtune (Vignali et al., 2020) to improve model performance (Merow et al., 2013) (Appendix S5).

We used the true skill statistics (TSS) and the area under the curve (AUC) of the receiver operating characteristic to evaluate model performance. We employed random cross‐validation to estimate the binary threshold for predictions and then assessed the model's performance with geographic block cross‐validation with the R package blockCV (Valavi et al., 2019). We combined the models generated from different algorithms into an ensemble model by averaging their outputs, weighing each model's contribution based on its AUC and TSS scores, and excluding models with low discrimination ability (AUC < 0.75 and TSS < 0.5). Final individual models were then run with all location records. Continuous predictions of the single models were then converted to binary presence–absence maps with the approach that maximizes the sum of model sensitivity and specificity (Liu et al., 2013).

We projected our models to 2041–2060 with the RCP 8.5 and RCP 4.5 emission scenarios and the 3 General Circulation Models (GCMs). We combined the outputs of models generated with the mean of the 3 GCMs. We calculated the extent of changes in range suitability between present and future conditions in biomod2 by overlapping the binary present and future model output maps for each bat species and calculating actual and percent changes in predicted range size (number of cells). Subsequently, the binary maps for each bat species were added to produce species richness maps, which we used to calculate changes in diversity under future climate change at each raster cell.

We ran a multivariate environmental similarity surfaces (MESS) analysis, as recommended by Elith et al. (2010), across the whole of Europe and North Africa to determine whether our study area was predicted to be in nonanalog condition. The MESS plots showed that all variables were within the range present in the training (current) data across Europe, with the exception of Bio4 in parts of Ukraine and Russia and Bio5 in the Sahara (Appendix S6). Neither of these areas was included in our projected models and subsequent analyses.

### Community‐level and FD analyses

To determine how climate change will affect bat community composition, we calculated beta diversity with the Jaccard dissimilarity index between present and future predictions at a 50‐km resolution. To compute the Jaccard dissimilarity index, we used the predicted presence and relative occupancy of species within each grid cell. The relative occupancy of species was calculated based on the proportion of the 50‐km cell predicted to be occupied by the species. We partitioned beta diversity into community turnover and nestedness in the betapart R package 1.6 (Baselga & Orme, 2012). This partition allows us to discriminate between community changes due to species substitutions (turnover) and community changes due to the loss of existing species (nestedness).

To assess the effects of climate change on FD, we assembled morphological and ecological trait data from a comprehensive data set for European bat traits (EuroBaTrait 1) (Froidevaux et al., 2023) that provides information on key dimensions of the bat niche and ecological attributes. We initially selected 13 key traits that are important for characterizing bats and have good coverage across the studied species. After removing highly correlated traits, the following 10 traits were retained: home range (area used to satisfy daily needs, representing movement behavior), estimated extent of occurrence (range size, representing extinction risk due to restricted geographic distribution), upper elevation limit, dietary diversity, dietary specialization index (range from specialists to generalists), body mass (influencing metabolic rate, energy demand, and diet), aspect ratio index (measure of wing morphology, relates to movement and dispersal behavior), echolocation call peak frequency (relates to foraging behavior and habitat use), foraging habitat selection, and thermal index (average temperatures across the species’ range) (trait descriptions and how they were measured are in Appendix S7). These traits relate to species foraging and movement ability, environmental tolerance, and energy demand and consumption and therefore represent how a given organism affects the community structure and species interaction network, with potential consequences for ecosystem functioning (Thévenin et al., 2022).

Because 10 of the 13 functional traits had missing data for some species (mean = 9.7 species, range 2–21, corresponding to a mean = 26.2%, range 5.4–56.7% of the total), we filled data gaps through imputation with missForest imputation (Stekhoven & Bühlmann, 2012; Stewart et al., 2023). missForest uses a random forest approach to impute missing values and effectively captures complex relationships among continuous and categorical variables. Previous comparative analyses have demonstrated good imputation performance (Penone et al., 2014). We included phylogenetic eigenvectors in the imputation by conducting a principal components analysis (PCA) on pairwise phylogenetic distances among species. The first 10 phylogenetic eigenvectors were used as predictor variables in the missForest imputation procedure, following Penone et al. (2014), alongside functional traits. Errors were assessed using the normalized root mean squared error (NRMSE):

(1) NRMSE=100×RMSE/rangeoftheoriginalvariable.

The imputation resulted in relatively low errors, which were deemed acceptable for the purpose of our analyses. The average NRMSE for the imputed values was 19.7% (range 14.4–25.1%).

Then, we used the predicted species presence and relative occupancy per 50‐km cell to calculate FD for the 2 time periods with the FD R package 1.0 (Laliberté et al., 2014). We used the functional dispersion metric, which represents the average distance of individual species to the weighted centroid of all species within a multidimensional trait space (Laliberte & Legendre, 2010). As for beta diversity, the weighting of functional dispersion was obtained using the predicted relative occupancy of species per grid cell with the SDM binary outputs.

We calculated the difference between present and future estimates of the FD metrics. To assess geographic patterns of trait distributions and predict their changes over time, we also estimated the community‐weighted mean (CWM) of each trait value per cell and measured its difference between the 2 time steps. The CWM is measured as the mean trait value for all species in the community, weighted by species relative abundance (in this case, their relative occupancy per 50‐km grid cell). We then explored each trait visually to identify changes in each trait.

To estimate the contribution of individual species’ range changes to the predicted change in functional dispersion, we fitted a random forest model. We used the predicted change in species occupancy per cell as a predictor variable and the predicted change in functional dispersion as the response variable. We fitted 1000 random trees and varied the number of variables randomly sampled as candidates at each split between 6 and 20. We selected 12 as the value resulting in the minimum error estimate.

## RESULTS

### Species distribution model performance and current patterns of range suitability

The average performance of the 4 algorithms was good (0.8) to excellent (>0.9), as indicated by the relatively high cross‐validation data mean AUC scores (full model: AUC = 0.905 [SD 0.037], range 0.831–0.971; Area Under the Curve cross‐validation (AUCcv) = 0.871 [0.016], range 0.798–0.9559) and TSS scores (full model: 0.720 [0.985], range 0.578–0.931; TSS cross‐validation 0.593 [0.037], range 0.383–0.819). Although the contribution of predictor variables in the model varied among species, overall, climatic variables contributed the most to the models of all bat species. The maximum temperature of the warmest month (BIO05) had the highest contribution (Appendix S8).

### Future projections

**FIGURE 1:**
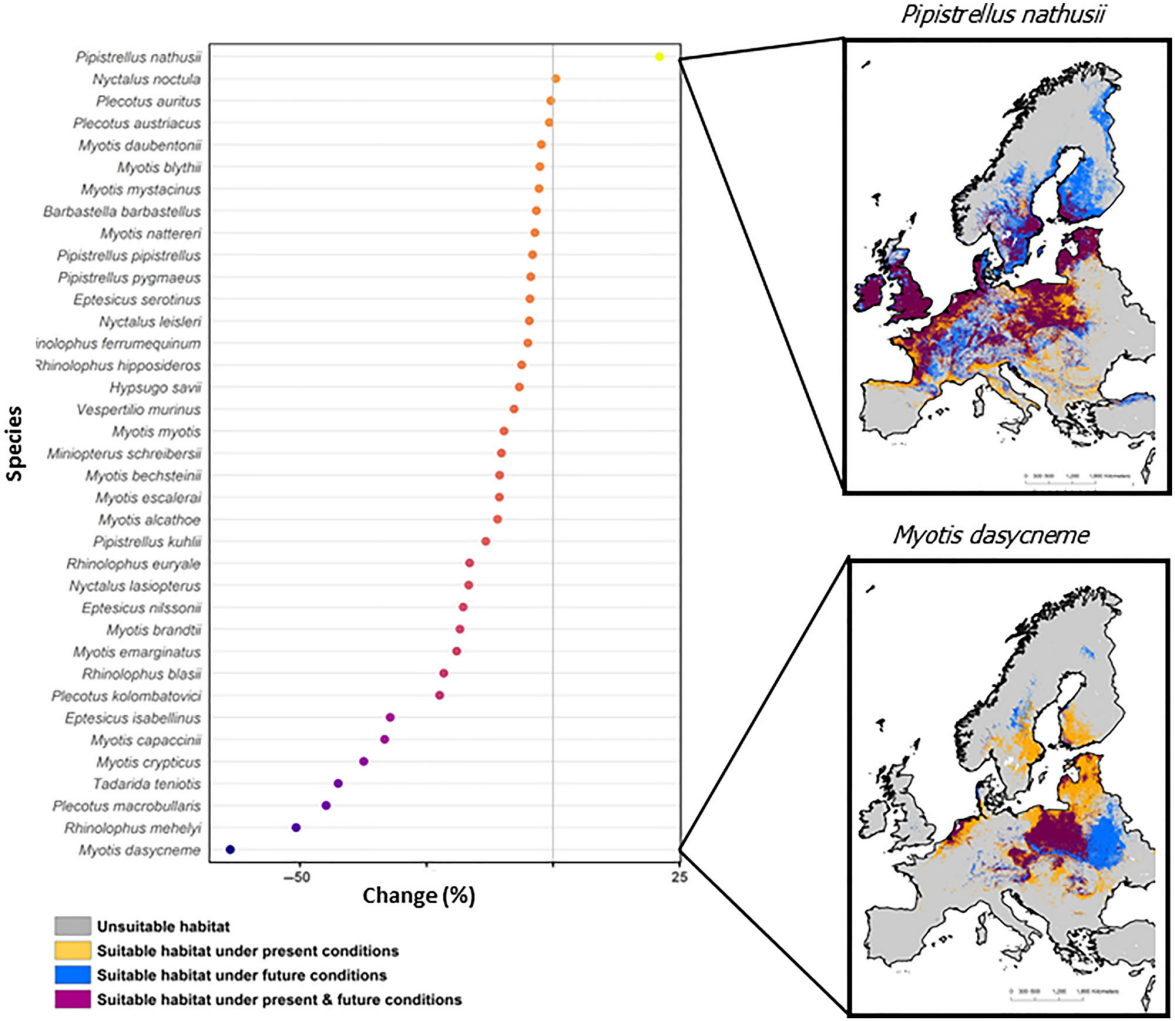
(a) Percent range change of each European bat species in the future under RCP (representative concentration pathway) 8.5 scenario for 2050 (0%, stable distribution; <0%, contracted distribution) and binary overlap map between current and future (2050) model predictions for (b) *Pipistrellus nathusii* (predicted to experience range gains) and (c) *Myotis dasycneme* (predicted to experience the greatest range losses).

Under future conditions (RCP 8.5), most European bat species were projected to experience range losses due to climate change (Figure 1). Projected range changes were more moderate under the moderate‐emission scenario (RCP 4.5) but followed very similar patterns (Appendix S9). Of the 37 modeled bat species, 7 were predicted to experience severe range contractions of >30% by 2050. The greatest losses of range were predicted for *Myotis dasycneme* (∼70%), followed by *R. mehelyi* (∼50%). Only one of the 37 European bat species, *Pipistrellus nathusii*, was predicted to experience range gains of approximately 21% under future climate change scenarios. *Nyctalus noctula*, *Plecotus auritus*, and *Plecotus austriacus* were predicted overall to experience small net changes in the extent of their range under climate change. Nonetheless, under climate change, the ranges of all 4 species were predicted to shift to higher latitudes. These range shifts were characterized by southern European range reductions and northern European gains. The ranges of the remaining 5 species, *Eptesicus isabellinus*, *Myotis capaccinii*, *Myotis crypticus*, *Tadarida teniotis*, and *Plecotus macrobullaris*, were predicted to contract by 30–45% (Figure 1; see https://doi.org/10.5061/dryad.2z34tmpx6 for details).

### Community turnover, species richness, and FD

**FIGURE 2:**
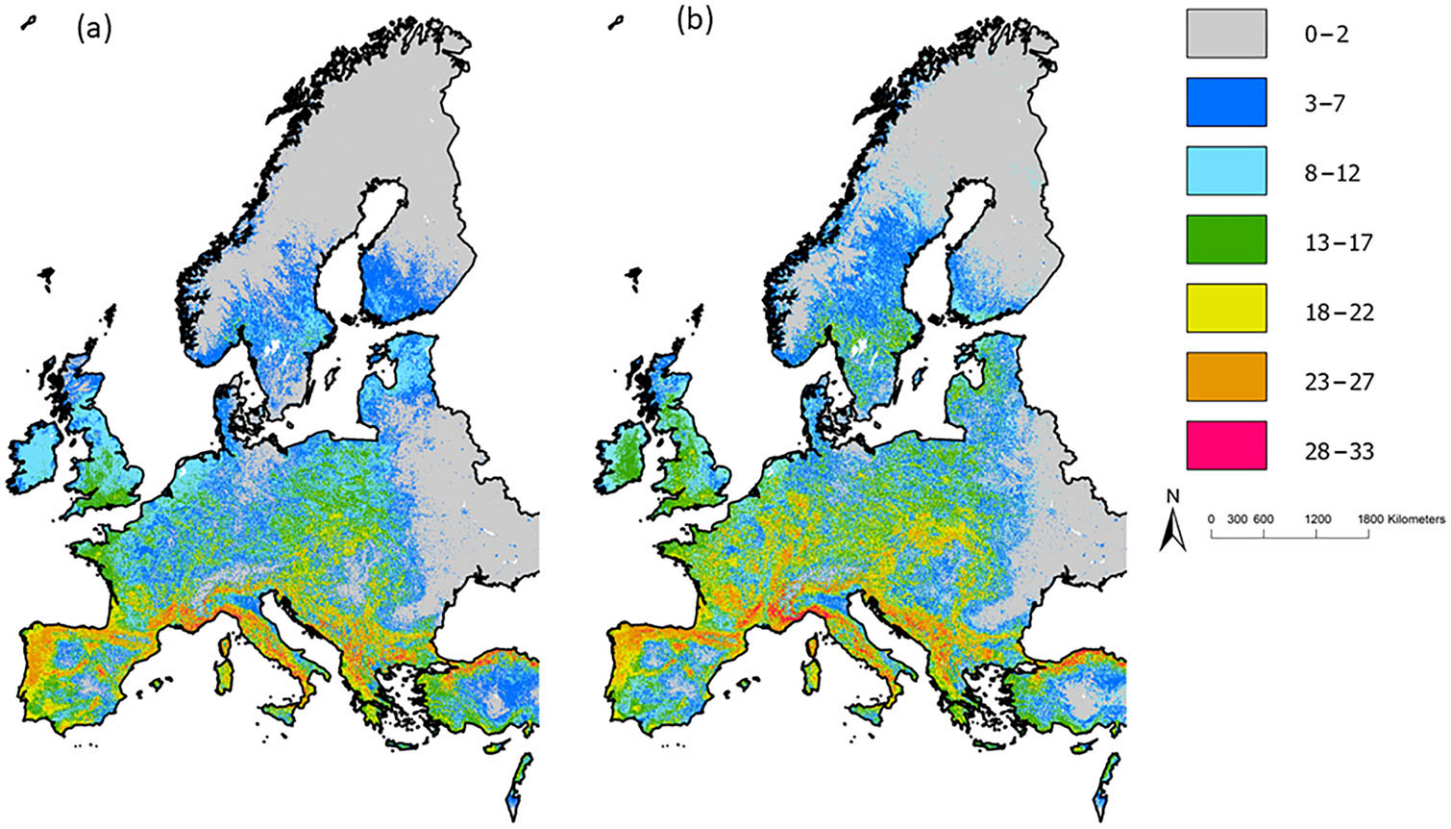
Modeled patterns of species richness of 37 European bats under (a) current and (b) future (RCP [representative concentration pathway] 8.5 scenario for 2050) climactic conditions.

Models for all 37 European bat species predicted range losses primarily in southern Europe, where the greatest number of species is currently found (up to 33 modeled species), and gains in northern latitudes. These shifts in geographical distributions altered patterns of diversity and community composition across Europe. Our models predicted an increase in species richness in Ireland, western Britain, and central and northern Europe. Many parts of southern Europe, such as the Iberian Peninsula, were predicted to experience a high negative turnover for the modeled species (Figure 2). Considerable losses in geographic range were predicted in southern Spain and the Mediterranean islands for some species, such as *E. isabellinus*, *P. pipistrellus*, and *Pipistrellus pygmaeus*.

**FIGURE 3:**
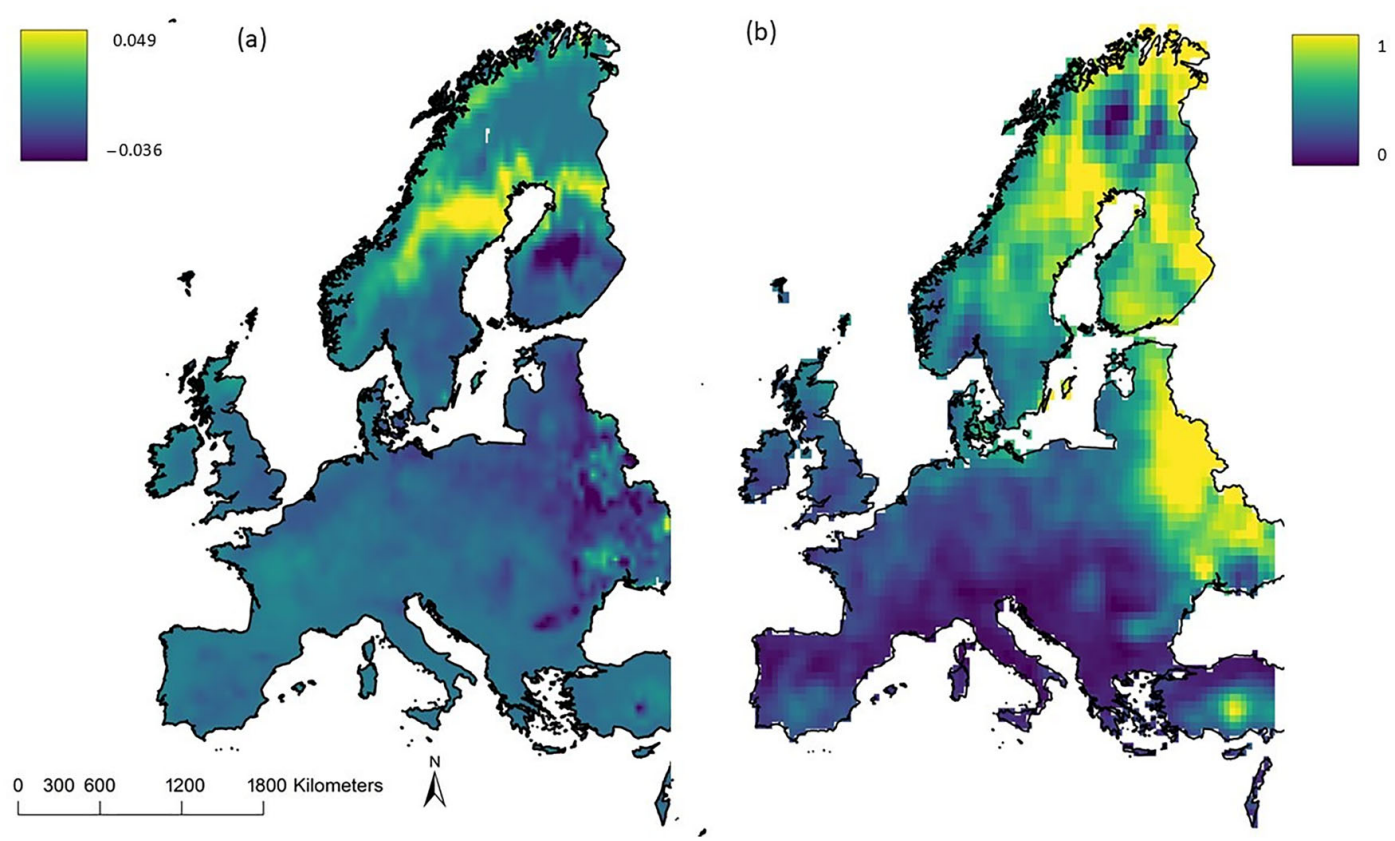
Predicted functional diversity (FD) and community turnover of 37 bat species across Europe currently and in 2050 under RCP (representative concentration pathway) 8.5 emissions scenario: (a) changes in functional diversity (Fdis) across 10 traits (values, absolute values; negative values, decrease in Fdis; positive values, increase in Fdis) and (b) community turnover (dark blue, no observed change in assemblage composition; yellow, change in assemblage composition).

**FIGURE 4:**
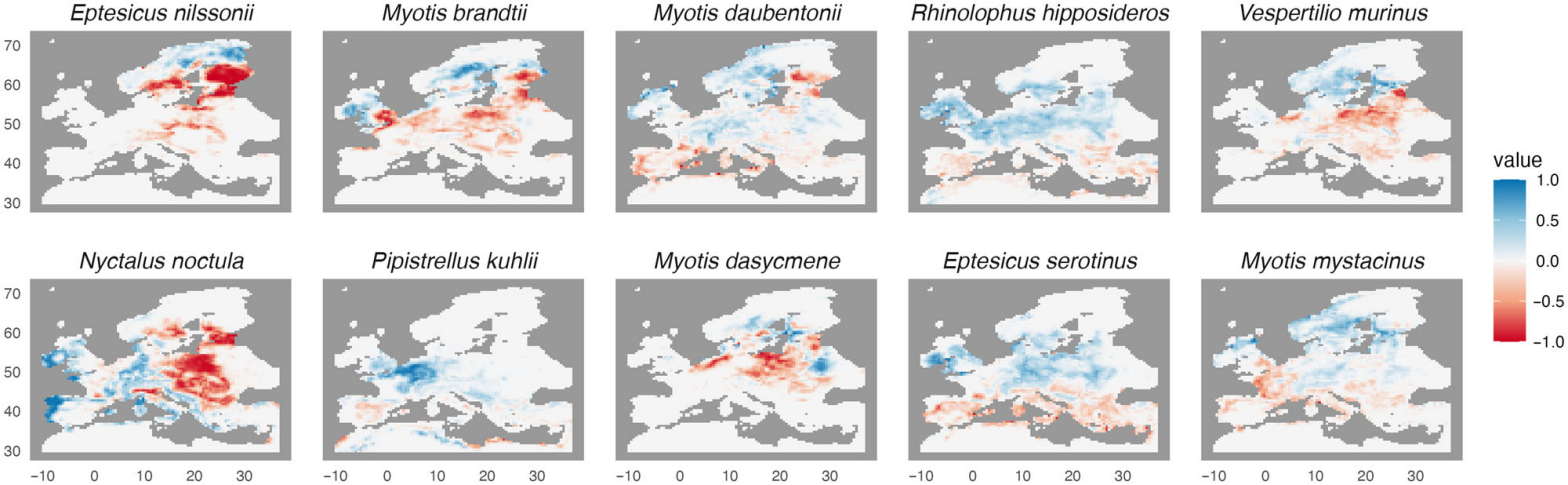
Predicted change in the distribution of the 10 species that contribute most to change in functional dispersion in order of their contribution (−1, 100% loss in 50 × 50‐km cells; +1, 100% gain in 50 × 50‐km cells).

Functional diversity followed a spatial pattern similar to that of community turnover; FD changes (gains and losses) were heterogeneous. Models projected large increases in FD in northern latitudes, especially in Norway, northern Sweden, and Finland (Figure 3). These increases aligned closely with substantial range shifts of, for example, *Myotis brandtii* and *Myotis mystacinus* to these higher latitudes (Figure 4). In contrast, certain areas in southern Sweden and Finland were predicted to experience a reduction in FD, despite an increase in species richness (Figure 2). These changes were primarily attributed to the loss of morphologically distinct species, such as *Eptesicus nilssonii* (Figure 4). In contrast, Eastern Europe, eastern England, and parts of southern Spain were predicted to face decreases in FD for the modeled species (Figure 3). These decreases were associated with range losses of *Vespertilio murinus*, *N. noctula*, and *M. mystacinus* (Figure 4).

The best‐supported model for the contribution of individual species range changes to the predicted change in functional dispersion explained 64.83% of the variance. The most important species driving the change in functional dispersion were *E. nilssonii* and *M. brandtii*, followed by *M. daubentonii*, *Rhinolophus hipposideros*, *V. murinus*, *N. noctula*, *P. kuhlii*, *M. dasycneme*, and *E. serotinus. Myotis mystacinus* also contributed, but to a lesser extent (Appendix S10).

**FIGURE 5:**
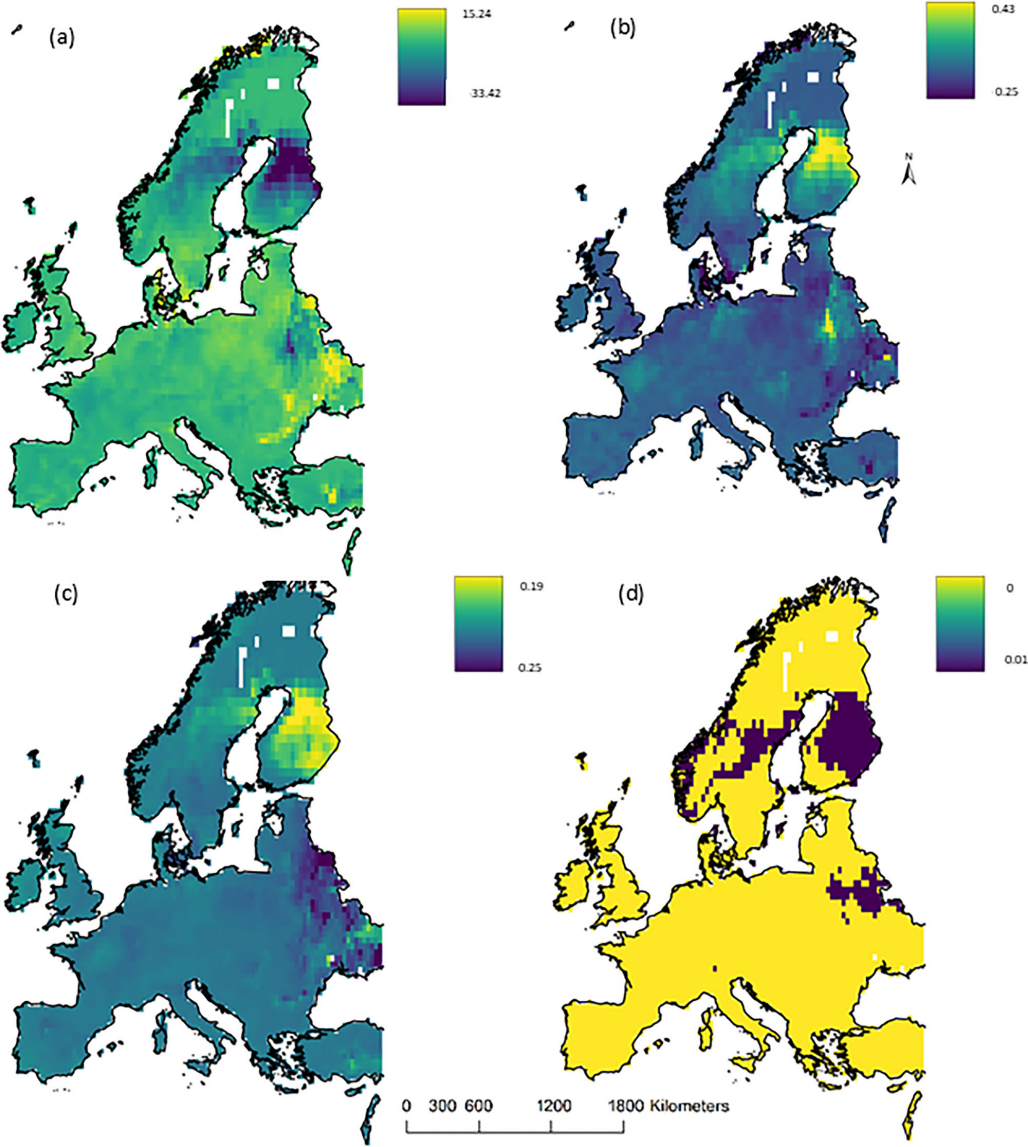
Predicted changes in modeled European bat species: (a) dietary specialization (dark blue, generalist species; yellow, specialist species), (b) diet diversity, (c) body mass, and (d) boreal forest foraging habitat (yellow, no association with boreal forest; dark blue, association with boreal forest; values in legends, absolute values for each range).

Maps of changes in each functional trait showed that the increase in FD across Fennoscandia was driven primarily by dietary traits, whereby diet diversity increased, while diet specialization decreased. We found an opposite trend in Eastern Europe, eastern Britain, Italy, and southern Spain, where the same dietary diversity trait decreased, resulting in a reduced FD (Figure 5). For the Fennoscandian region, the FD increase was also closely associated with body mass and thermal index traits. In contrast, in Spain and Italy, the thermal index trait appeared to be associated with the observed decrease in FD. However, in Finland, the decline in FD was closely associated with a reduction in species associated with boreal forests. Finally, in Eastern Europe, the predicted decrease in FD was predominantly linked to aspect ratio and body mass traits (Figure 5).

## DISCUSSION

We assessed the potential impacts of climate change on the distribution, composition, and FD of bats at a continental scale. Our results predicted substantial shifts in range and FD across Europe, with notable range contractions for most bat species. Range contractions were predicted to occur in southern Europe, as European bats experience an overall shift in climatic suitability toward higher latitudes. These changes align with broader biodiversity trends and climate change predictions (Bilgin et al., 2012; Maclean & Wilson, 2011; Maiorano et al., 2011; Parmesan & Yohe, 2003; Rebelo et al., 2010). However, ours is the first study to relate such shifts to changes in composition and FD.

### Predicted changes in species’ range

Climate change is predicted to lead to the extinction of up to 10% of all European nonvolant mammals within the next century, with as many as 25% predicted to become critically endangered (Levinsky et al., 2007). A similar fate could affect bats in Europe because two thirds of the species we analyzed are already classified as endangered, vulnerable, or near threatened (IUCN, 2022). In our study, the predicted range losses for 33 out of 37 bat species by 2050 could cause population declines, increasing their vulnerability to loss of genetic diversity and evolutionary potential (Willi et al., 2006). Our predictions may be optimistic, given that bats may not be able to track their climatic niche at the pace of ongoing climate change due to their slow population growth rates, high philopatry, and long lifespans (Devictor et al., 2008). Even minor losses of habitat can lead to the extinction of local populations (McCarty, 2001), particularly when the species’ ability to colonize and establish in new suitable areas is limited by dispersal barriers (García‐Mudarra et al., 2009; Razgour, 2015), competition between species (Hall et al., 2016; Razgour et al., 2018; Salinas‐Ramos et al., 2021, 2020; Smeraldo et al., 2021), food availability (Krauel et al., 2015), roost requirements (Loeb & Winters, 2013), or habitat fragmentation (Frey‐Ehrenbold et al., 2013).

We found that most Mediterranean bat species were predicted to experience extensive range reductions. This is consistent with the findings of Rebelo et al. (2010) but contrasts with evidence of range expansion of some Mediterranean heat‐tolerant species, such as *P. kuhlii*, over the last 2 decades (Ancillotto et al., 2016). However, despite the overall predicted ∼10% reduction in *P. kuhlii*’s range, range losses were predicted in southern Europe (especially Iberian and Italian Peninsulas) and parts of North Africa, whereas range was predicted to increase in more northern latitudes. This is in line with the trend observed over the past few decades, whereby *P. kuhlii* has been expanding its range into central and Eastern Europe (Sachanowicz et al., 2006). In contrast, range losses in southern Europe have not been recorded yet, possibly because they are more challenging to confirm than range expansions. Predicted range losses in southern Europe are of particular concern because the Iberian, Italian, and Balkan Peninsulas have supported bat populations over thousands of years, while more northern parts of Europe were covered by ice sheets and arctic tundra during Pleistocene glaciation events (e.g., *B. barbastellus* [Rebelo et al., 2012] and the *Myotis nattereri* species complex [Salicini et al., 2013]). As a result, bat populations in southern Europe tend to have higher levels of genetic diversity (e.g., Razgour et al., 2013) and, in turn, greater evolutionary potential and ability to adapt and respond to environmental change and diseases (Hoban et al., 2022). Mediterranean species, such as *R. mehelyi*, *T. teniotis*, *M. capaccinii*, and *E. isabellinus*, were predicted to experience the largest range contractions, 25–50%. This contraction poses a severe threat to their ability to respond to global change and consequently to their survival. Considering that *R. mehelyi* is already listed as endangered on the International Union for Conservation of Nature Red List (Russo & Cistrone, 2023a), climate change poses additional stress to this species, increasing the likelihood of extirpation and extinction. This is a concern also for other Mediterranean species classified as vulnerable, *M. capaccinii* (Russo & Cistrone, 2023b) and *Rhinolophus blasii* (Russo & Cistrone, 2023c).

The predicted impact of climate change extended beyond Mediterranean species, significantly affecting those in northern latitudes as well (Jetz et al., 2007; Kerr & Packer, 1998; Sala et al., 2000; Virkkala et al., 2008). Temperature increases are expected to be more pronounced in high latitudes (Meehl et al., 2007), potentially leading to the loss of suitable climatic conditions. This is evident in our projections of ∼70% (range across models: 57–70%) range reduction, the highest projected in our study, for *M. dasycneme*, a species found in northern and central Europe that is classified as vulnerable (Russo & Cistrone, 2023). A similar trend is predicted for *E. nilssonii* (18% range loss; range across models 13–21%). In contrast, *M. brandtii* and *M. mystacinus* were predicted to expand their ranges to northern latitudes despite a decrease in their overall ranges.

The predicted northward range expansion of some bat species may be limited by short summer nights, although we did not model this. In Fennoscandia, photoperiods change dramatically throughout the summer and extend to continuous daylight or brief periods of white nights that are still very bright (Speakman et al., 2000). *Myotis mystacinus* and *M. brandtii* are highly sensitive to light, emerging later than other bat species and avoiding flying in open spaces (Jones & Rydell, 1994; Spoelstra et al., 2017; Suominen et al., 2023). Adverse weather conditions at northern latitudes and short summer nights can cause a mismatch between energy expenditure and foraging opportunities, which can constraint the northern range expansion of many bat species (Fjelldal et al., 2023).

Not all European bat species were predicted to experience range losses under climate change. Some species were predicted to either maintain their current ranges or experience range expansions. The diverse responses of bat species to climate change can be attributed to a variety of factors, including ecological flexibility and differences in diet, roosting requirements, and physiological and reproductive strategies, which are likely to influence their resilience to changing climatic conditions (Loeb & Winters, 2013; Sherwin et al., 2013). The only species predicted to experience a substantial range increase was *P. nathusii*, a highly adaptive species (Lundy et al., 2010). This species has moved into novel habitats as a result of increasing temperatures, shifting its range in response to recent climate change at a European scale (Blomberg, Vasko, Salonen, et al., 2021; Lundy et al., 2010). Because *P. nathusii* is a long‐distance migrant (Fleming et al., 2003), it can more easily reach new climatically suitable areas across Europe. Our results highlight the importance of considering species’ ecological and physiological traits to better understand the variability in their sensitivity to climate change.

### Changes in patterns of species richness

Our models predicted noticeable changes in patterns of bat species richness across Europe under climate change. Currently, areas with high bat species richness are predominantly found in southern Europe, particularly around the Mediterranean basin. Our models predicted a decrease in species richness in these areas by 2050 and a corresponding increase in more northern latitudes. Similar trends were projected in previous modeling studies for other European mammals (Levinsky et al., 2007) and birds (Austin & Rehfisch, 2005; Huntley et al., 2008). We modeled only species that currently occur in Europe, so species losses in southern Europe could be compensated for by species expanding their ranges from North Africa and the Middle East. The future range contractions and expansions predicted here and the possible arrival of new species not currently occurring in Europe will create novel species assemblages, which could contribute to high spatiotemporal species turnover in future communities, resulting in new biotic interactions, such as competition (Blois et al., 2013) or host–parasite/host–pathogen interactions (Carlson et al., 2022).

The observed northward shifts in European Odonata and Lepidoptera due to warming (Hickling et al., 2005; Parmesan et al., 1999) suggest a potential increase in invertebrate diversity, which can support the predicted increased diversity and northward range shifts of insectivorous bats. This parallel trend underscores the interconnectedness of species range shifts in response to climate change. Nevertheless, the global decline in insect abundance and diversity (Forister et al., 2019) represents a significant ecological challenge for bat species, one that affects their primary food resources irrespective of their geographic distribution.

### Changes in FD

The impact of climate change on patterns of FD has been understudied, especially in mammals (Thuiller et al., 2004). The predicted shifts in the distribution of suitable conditions for species under climate change are likely to drive changes in community structure and functional dynamics of bat populations across Europe, leading to functional changes (Salinas‐Ramos et al., 2020). We predicted an increased functional dispersion in Northern Europe, apart from southern Finland, and decreases in Eastern Europe and parts of southern Spain. In line with our predictions, these trends were predominantly driven by a noticeable community turnover in northern and eastern parts of Europe, an increase in species richness in northern latitudes, and a decrease in southern Europe.

Predicted changes in range were responsible for functional dispersion change in *E. nilssonii*, *M. brandtii*, and *N. noctula* in Scandinavia; in *M. brandtii*, *V. murinus*, *N. noctula*, and *M. dasycneme* in Eastern Europe; and in *P. kuhlii*, *M. brandtii*, and *R. hipposideros* in northern Central Europe and the United Kingdom. Biodiversity is often linked to enhanced ecosystem functioning due to its presumed positive relationship with FD (Hooper et al., 2005). However, the relationship between species and FD can vary among ecosystems and functions different species perform (Cadotte et al., 2011; Liira & Jürjendal, 2023). As such, an increase in species diversity does not necessarily lead to improved ecosystem services and functioning. The trend of increasing species richness alongside a decrease in functional dispersion that we found in southern Finland and the eastern part of Europe suggests that although new functional types may be moving northward, the bulk of species arriving in those areas may be functionally similar or redundant. In contrast, species lost from these areas, such as *E. nilssonii*, *M. brandtii*, and *N. noctula*, represent the loss of unique traits, including larger body size and association with boreal forests. This implies that the functional advantages of increased biodiversity from expanding bat species in Europe may be limited, as has been seen in fish and bird communities (Barbet‐Massin & Jetz, 2015; Pawluk et al., 2022). Functional redundancy (i.e., the similarity in functional traits among species [Walker, 1992]) is vital for ecosystem stability and resilience (Biggs et al., 2020). Acting as a buffer against the loss of species diversity, it helps maintain FD and is akin to an insurance policy for ecosystems (Gallagher et al., 2013; Gladstone‐Gallagher et al., 2019). This redundancy supports ecosystem resilience under changing climates (Kahmen et al., 2005) and suggests that an increase in species diversity, coupled with stable or reduced functional dispersion, can lead to robust and resilient ecosystem services.

Of the functional traits we included in our analyses, our models predicted changes in the dietary traits of European bat species, which has implications for their ecological roles. In Fennoscandia, we predicted an increase in dietary diversity and niche breadth, coupled with a reduction in diet specialization. Contrasting trends were predicted for Eastern Europe, the eastern United Kingdom, Italy, and southern Spain. These changes in dietary traits and trait structures, driven by climate change, not only signify ecological shifts but also reflect potential impacts on ecosystem functioning (Schneider et al., 2016) and eventual predator–prey mismatch that could affect species survival (Thackeray et al., 2016).

The decline in bat FD in southern Finland can be attributed to the decline in species associated with boreal forests and species with more specialist diets. Climate change will change the tree species composition in southern Finland. Broadleaved deciduous tree species will replace Norway spruce, which will change the properties of forest habitats (Kellomäki et al., 2005). This poses a direct threat to bat species that rely on these forests for roosting, foraging, and breeding and could lead to a decline in their populations in these regions. Our functional analysis predicted that this community will be replaced by a community of bats with traits that are better matched to the new forest environment.

In Fennoscandia, the observed increase in FD among bats is attributed to, for example, large body mass. Such traits are generally associated with enhanced adaptability to varying climatic conditions (Pacifici et al., 2017). In contrast, Eastern Europe was predicted to see an increase in bats with small body mass, suggesting a potential reduction in adaptability to climate change (Mundinger et al., 2021; Salinas‐Ramos et al., 2021). However, this observation in Eastern Europe should be interpreted with caution due to the lower density of records in some countries in this region. Nonetheless, these contrasting trends tend to reflect how different regions may experience varied impacts of climate change on bat physiology and, consequently, their ecological roles.

The overall predicted decrease in FD among bats is likely to have far‐reaching negative consequences for other species and their associated ecosystem services. This is a critical concern. Because bats play a pivotal role in maintaining ecological balance through their functions, such as pest control (Kunz et al., 2011), their decline could lead to cascading ecological effects (Frick et al., 2020; Schmitt et al., 2021). This could also affect agriculture productivity, influence forest ecosystem functioning, and further increase reliance on chemical pesticides that harm the environment and threaten human health (Frank, 2024; Rani et al., 2021). The predicted impacts of the loss or gain of morphologically distinct species, such as *E. nilssonii* and *R. mehelyi*, in southern Finland and southern Spain, respectively, illustrate the functional importance of these species in their ecosystems. The decrease in FD of the local bat community may result in decreased diversity of the impacts of bats on ecosystems, including reduced top‐down effects on different insect taxa because of a less diverse diet, and more restricted influence of bats on fewer habitat types. Moreover, a lower FD could lead to lower resilience to environmental change (Elmqvist et al., 2003). Given these potential consequences, it is important to understand which functional traits make bats particularly sensitive to climate change and how bat communities can be managed to retain key functions.

### Conservation implications

Our findings underscore the urgent need for proactive and adaptive conservation strategies and coordinated international monitoring efforts to track bat responses to climate change at the continental scale. The results are being used to inform the development of a continental‐scale monitoring network to track bat responses to climate change. The predicted northward shift in suitable conditions, coupled with the risks faced by species in southern Europe, presents a complex challenge that requires a multifaceted approach to conservation. Conservation efforts should focus on protecting and enhancing habitats and roosts in newly suitable areas and current ranges. Artificial roost creation may be needed in new areas that are climatically suitable, such as Fennoscandia, where underground natural roosts are limited (Blomberg, Vasko, Meierhofer, et al., 2021). In addition, there is a need for landscape‐scale management strategies to increase landscape connectivity to facilitate range shifts under climate change by providing green corridors and reducing barriers to movement. Management strategies should take into account the complex interplay of ecological, climatic, and geographic factors that affect each species. We demonstrate the need to consider FD in conservation by targeting species with key functional traits, such as large body size and high dietary diversity, which are predicted to be lost in areas with high predicted losses in FD under climate change, such as southern Spain and parts of eastern Europe. This holistic approach is key not only for preserving bat populations but also for maintaining the critical ecosystem services bats provide.

## AUTHOR CONTRIBUTIONS

*Conceptualization*: Orly Razgour, Danilo Russo, and Hugo Rebelo. *Study design and methodology*: Orly Razgour, Luca Santini, Hugo Rebelo, Roberto Novella‐Fernandez, and Penelope C. Fialas. *Running models*: Penelope C. Fialas, Francisco Amorim, Francisco Marques, and Roberto Novella‐Fernandez. *Formal analysis and investigation*: Penelope C. Fialas and Luca Santini. *Data collection*: Orly Razgour and Adi Domer. *Writing and original draft preparation*: Penelope C. Fialas. All authors provided location records and contributed to manuscript revisions.

## Supporting information




**Appendix S1**. Overview of the study extent and the occurrence records obtained through the European COST ACTION ClimBats network for the 37 bat species across Europe, North Africa and eastern parts of the Mediterranean basin.
**Appendix S2**. List of publication obtained location records from.
**Appendix S3**. Number of location records per bat species
**Appendix S4**. Initial environmental variables used for the species distribution modelling. Bioclimatic variable in red was removed because it was highly correlated.
**Appendix S5**. Parameters tuned for each species using “SDMtune” R package.
**Appendix S7**. Definitions of functional traits used in the study. Traits in red were removed from the analysis because they were highly correlated.
**Appendix S6**. Multivariate Environmental Similarity Surfaces (MESS) plots. a) MESS output map showing areas across Europe and North Africa where environmental variables are outside the range present in the training data. Red represents areas where one or more variables are outside their training range. b) MESS output map showing the most dissimilar variables outside their training range. Orange colours show that none of the variables are outside their training range.
**Appendix S8**. Sankey diagram showing the contribution of the environmental variables to the species distribution models for each bat species. The width of the lines represents the strength or weight of the contribution of each variable to the model for each species.
**Appendix S9**. Predicted range suitability for 37 European bat species under current and future conditions (RCP 4.5 and RCP 8.5 emission scenarios) based on ensemble modeling.
**Appendix S10**. Random forest variable importance estimates indicating the relative contribution of the 37 bat species to the change in functional diversity.
**Figure S2**. Methodological framework applied to predict the impact of climate change on range suitability, species richness, community composition and functional diversity for 37 European bat species. The blue boxes are the results of the analyses.
