# Supplementary material for: Changes in community composition and functional diversity of European bats under climate change
Source: Conserv Biol. 2025 Apr 1;39(4):e70025. doi: 10.1111/cobi.70025 (PMC12309660; doi:10.1111/cobi.70025)
Supplement: Supplementary file 1 — Appendix S1. Overview of the study extent and the occurrence records obtained through the European COST ACTION ClimBats network for the 37 bat species across Europe, North Africa and eastern parts of the Mediterranean basin. Appendix S2. List of publication obtained location records from. Appendix S3. Number of location records per bat species Appendix S4. Initial environmental variables used for the species distribution modelling. Bioclimatic variable in red was removed because it was highly correlated. Appendix S5. Parameters tuned for each species using “SDMtune” R package. Appendix S7. Definitions of functional traits used in the study. Traits in red were removed from the analysis because they were highly correlated. Appendix S6. Multivariate Environmental Similarity Surfaces (MESS) plots. a) MESS output map showing areas across Europe and North Africa where environmental variables are outside the range present in the training data. Red represents areas where one or more variables are outside their training range. b) MESS output map showing the most dissimilar variables outside their training range. Orange colours show that none of the variables are outside their training range. Appendix S8. Sankey diagram showing the contribution of the environmental variables to the species distribution models for each bat species. The width of the lines represents the strength or weight of the contribution of each variable to the model for each species. Appendix S9. Predicted range suitability for 37 European bat species under current and future conditions (RCP 4.5 and RCP 8.5 emission scenarios) based on ensemble modeling. Appendix S10. Random forest variable importance estimates indicating the relative contribution of the 37 bat species to the change in functional diversity. Figure S2. Methodological framework applied to predict the impact of climate change on range suitability, species richness, community composition and functional diversity for 37 European bat species. Th [file COBI-39-e70025-s001.docx]

**Supporting Material**

**Changes in community composition and functional diversity of European bats under climate change**

**Appendix S2.** List of publication obtained location records from.

| Location Records | Literature |
| --- | --- |
| Algeria |  |
| Libya | Benda, P., Spitzenberger, F., Hanák, V., Andreas, M., Reiter, A., Ševčík, M., ... & Uhrin, M. (2014). Bats (Mammalia: Chiroptera) of the Eastern Mediterranean and Middle East. Part 11. On the bat fauna of Libya II. *Acta Societatis Zoologicae Bohemicae*, *78*, 1-162. |
| Syria | Benda, P., Andreas, M., Kock, D., Lucan, R. K., Munclinger, P., Nova, P., ... & Weinfurtova, D. (2006). Bats(Mammalia: Chiroptera) of the Eastern Mediterranean. Part 4. Bat fauna of Syria: distribution, systematics, ecology. *Acta Societatis Zoologicae Bohemicae*, *70*(1), 1-329. |
| Lebanon | Benda, P., Abi Said, M. R., Bou Jaoude, I., Karanouh, R., Lučan, R. K., Sadek, R., ... & Horáček, I. (2016). Bats (Mammalia: Chiroptera) of the Eastern Mediterranean and Middle East. Part 13. Review of distribution and ectoparasites of bats in Lebanon. *Acta Societatis Zoologicae Bohemicae*, *80*, 207-316. |
| Egypt | Benda, P., Dietz, C., Andreas, M., Hotový, J., Lučan, R. K., Maltby, A., ... & Vallo, P. (2008). Bats (Mammalia: Chiroptera) of the Eastern Mediterranean and Middle East. Part 6. Bats of Sinai (Egypt) with some taxonomic, ecological and echolocation data on that fauna. *Acta Societatis Zoologicae Bohemicae*, *72*(1-2), 1-103. |
| Morocco | Benda, P., Ruedi, M., & Aulagnier, S. (2004). New data on the distribution of bats (Chiroptera) in Morocco. *Vespertilio*, *8*, 13-44. |
| Poland | Atlas Ssaków Polski" ([https://www.iop.krakow.pl/ssaki](https://eur03.safelinks.protection.outlook.com/?url=https%3A%2F%2Fwww.iop.krakow.pl%2Fssaki&data=05%7C02%7Cpf313%40exeter.ac.uk%7C346bba9dba864dd3db9408dd574cf7d8%7C912a5d77fb984eeeaf321334d8f04a53%7C0%7C0%7C638762710263723398%7CUnknown%7CTWFpbGZsb3d8eyJFbXB0eU1hcGkiOnRydWUsIlYiOiIwLjAuMDAwMCIsIlAiOiJXaW4zMiIsIkFOIjoiTWFpbCIsIldUIjoyfQ%3D%3D%7C0%7C%7C%7C&sdata=0TTJXw2%2F4LgOQOacRS71RXq5yfk0tQD5OfcmWpC%2FxSA%3D&reserved=0)) (Copyright © Instytut Ochrony Przyrody PAN, 2010. Projekt i realizacja strony: Edward Bobeł HTML, CSS; Wiesław Król ASP.NET, SQL) |

**Appendix S3.** Number of location records per bat species

| Species name | Number of location records |
| --- | --- |
| *Barbastella barbastellus* | 438 |
| *Eptesicus isabellinus* | 168 |
| *Eptesicus nilssonii* | 474 |
| *Eptesicus serotinus* | 751 |
| *Hypsugo savii* | 347 |
| *Myotis alcathoe* | 101 |
| *Myotis bechsteinii* | 235 |
| *Myotis blythii* | 303 |
| *Myotis brandtii* | 253 |
| *Myotis capaccinii* | 206 |
| *Myotis crypticus* | 93 |
| *Myotis dasycneme* | 188 |
| *Myotis daubentonii* | 881 |
| *Myotis emarginatus* | 405 |
| *Myotis escalerai* | 141 |
| *Myotis myotis* | 571 |
| *Myotis mystacinus* | 443 |
| *Myotis nattereri* | 334 |
| *Miniopterus schreibersii* | 514 |
| *Nyctalus lasiopterus* | 93 |
| *Nyctalus leisleri* | 464 |
| *Nyctalus noctula* | 593 |
| *Plecotus auritus* | 742 |
| *Plecotus austriacus* | 486 |
| *Plecotus kolombatovici* | 62 |
| *Pipistrellus kuhlii* | 596 |
| *Plecotus macrobullaris* | 106 |
| *Pipistrellus nathusii* | 495 |
| *Pipistrellus pipistrellus* | 929 |
| *Pipistrellus pygmaeus* | 522 |
| *Rhinolophus blasii* | 125 |
| *Rhinolophus euryale* | 351 |
| *Rhinolophus ferrumequinum* | 705 |
| *Rhinolophus hipposideros* | 679 |
| *Rhinolophus mehelyi* | 200 |
| *Tadarida teniotis* | 302 |
| *Vespertilio murinus* | 247 |

**Appendix S4.** Initial environmental variables used for the species distribution modelling. Bioclimatic variable in red was removed because it was highly correlated.

| Variable | Description |
| --- | --- |
| CHELSA_bio10_04 | Temperature Seasonality |
| CHELSA_bio10_05 | Max Temperature of Warmest Month |
| CHELSA_bio10_11 | Mean Temperature of Coldest Quarter |
| CHELSA_bio10_12 | Annual Precipitation |
| CHELSA_bio10_15 | Precipitation Seasonality |
| CHELSA_bio10_18 | Precipitation of Warmest Quarter |
| LC_Forested | Land Cover - Forest |
| LC_urban | Land Cover - Urban |
| rugness_bi | Ruggedness index |

**Appendix S5.** Parameters tuned for each species using “SDMtune” R package.

| Species name | ANN_size | ANN_decay | ANN_rang | ANN_maxit | BRT_distribution | BRT_n.trees | BRT_interaction.depth | BRT_shrinkage | BRT_bag.fraction | Maxent_fc | Maxent_reg | Maxent_iter | GAM_k |
| --- | --- | --- | --- | --- | --- | --- | --- | --- | --- | --- | --- | --- | --- |
| Barbastella barbastellus | 20 | 0.5 | 0.7 | 1000 | bernoulli | 740 | 4 | 0.06 | 0.5 | lq | 3.74 | 500 | 4 |
| Eptesicus isabellinus | 7 | 0.05 | 0.7 | 500 | bernoulli | 640 | 3 | 0.09 | 0.5 | l | 1.76 | 500 | 4 |
| Eptesicus nilssonii | 61 | 0.5 | 0.7 | 1000 | bernoulli | 820 | 2 | 0.07 | 0.5 | lq | 1.02 | 500 | 4 |
| Eptesicus serotinus | 22 | 0.1 | 0.7 | 1000 | bernoulli | 820 | 4 | 0.05 | 0.5 | lq | 1.02 | 500 | 4 |
| Hypsugo savii | 52 | 0.05 | 0.7 | 100 | bernoulli | 380 | 4 | 0.09 | 0.5 | lq | 1.02 | 500 | 4 |
| Myotis alcathoe | 48 | 0.1 | 0.7 | 1000 | bernoulli | 1000 | 4 | 0.07 | 0.5 | lq | 1.02 | 500 | 4 |
| Myotis bechsteinii | 50 | 0.05 | 0.7 | 1000 | bernoulli | 640 | 3 | 0.05 | 0.5 | lq | 1.02 | 500 | 4 |
| Myotis blythii | 25 | 0.3 | 0.7 | 1000 | bernoulli | 340 | 2 | 0.07 | 0.5 | lq | 1.02 | 500 | 4 |
| Myotis brandtii | 20 | 0.5 | 0.7 | 1000 | bernoulli | 640 | 3 | 0.05 | 0.5 | lq | 1.98 | 500 | 4 |
| Myotis capaccinii | 40 | 0.5 | 0.7 | 1000 | bernoulli | 400 | 3 | 0.09 | 0.5 | lq | 2.24 | 500 | 4 |
| Myotis crypticus | 48 | 0.5 | 0.7 | 1000 | bernoulli | 280 | 4 | 0.08 | 0.5 | lq | 2.88 | 500 | 4 |
| Myotis dasycneme | 79 | 0.1 | 0.7 | 500 | bernoulli | 200 | 3 | 0.05 | 0.5 | lq | 1.02 | 500 | 4 |
| Myotis daubentonii | 20 | 0.5 | 0.7 | 1000 | bernoulli | 460 | 4 | 0.06 | 0.5 | lq | 1.02 | 500 | 4 |
| Myotis emarginatus | 20 | 0.05 | 0.7 | 1000 | bernoulli | 640 | 4 | 0.09 | 0.5 | lq | 1.02 | 500 | 4 |
| Myotis escalerai | 60 | 0.3 | 0.7 | 500 | bernoulli | 400 | 4 | 0.08 | 0.5 | lq | 1.02 | 500 | 4 |
| Myotis myotis | 16 | 0.1 | 0.7 | 1000 | bernoulli | 640 | 4 | 0.07 | 0.5 | lq | 4.04 | 500 | 4 |
| Myotis mystacinus | 52 | 0.5 | 0.7 | 500 | bernoulli | 380 | 4 | 0.09 | 0.5 | lq | 1.02 | 500 | 4 |
| Myotis nattereri | 20 | 0.5 | 0.7 | 1000 | bernoulli | 1020 | 3 | 0.05 | 0.5 | lq | 1.02 | 500 | 4 |
| Miniopterus schreibersii | 11 | 0.01 | 0.7 | 1000 | bernoulli | 640 | 4 | 0.05 | 0.5 | lq | 1.02 | 500 | 4 |
| Nyctalus lasiopterus | 61 | 0.5 | 0.7 | 1000 | bernoulli | 400 | 2 | 0.09 | 0.5 | lq | 1.02 | 500 | 4 |
| Nyctalus leisleri | 20 | 0.5 | 0.7 | 1000 | bernoulli | 200 | 3 | 0.06 | 0.5 | lq | 1.86 | 500 | 4 |
| Nyctalus noctula | 22 | 0.5 | 0.7 | 1000 | bernoulli | 1000 | 3 | 0.07 | 0.5 | lq | 1.02 | 500 | 4 |
| Plecotus auritus | 18 | 0.05 | 0.7 | 1000 | bernoulli | 820 | 4 | 0.05 | 0.5 | lq | 1.02 | 500 | 4 |
| Plecotus austriacus | 33 | 0.05 | 0.7 | 500 | bernoulli | 960 | 4 | 0.05 | 0.5 | lq | 3.44 | 500 | 4 |
| Plecotus kolombatovici | 78 | 0.5 | 0.7 | 1000 | bernoulli | 400 | 1 | 0.07 | 0.5 | lq | 4.6 | 500 | 4 |
| Pipistrellus kuhlii | 7 | 0.05 | 0.7 | 1000 | bernoulli | 640 | 4 | 0.09 | 0.5 | lq | 1.02 | 500 | 4 |
| Plecotus macrobullaris | 61 | 0.3 | 0.7 | 1000 | bernoulli | 200 | 3 | 0.06 | 0.5 | lq | 4.6 | 500 | 4 |
| Pipistrellus nathusii | 48 | 0.3 | 0.7 | 1000 | bernoulli | 820 | 4 | 0.05 | 0.5 | lq | 2.56 | 500 | 4 |
| Pipistrellus pipistrellus | 18 | 0.1 | 0.7 | 1000 | bernoulli | 640 | 4 | 0.09 | 0.5 | lq | 1.02 | 500 | 4 |
| Pipistrellus pygmaeus | 20 | 0.5 | 0.7 | 500 | bernoulli | 520 | 4 | 0.05 | 0.5 | lq | 1.02 | 500 | 4 |
| Rhinolophus blasii | 70 | 0.3 | 0.7 | 1000 | bernoulli | 820 | 4 | 0.09 | 0.5 | lq | 1.02 | 500 | 4 |
| Rhinolophus euryale | 6 | 0.01 | 0.7 | 100 | bernoulli | 860 | 3 | 0.07 | 0.5 | lq | 3.26 | 500 | 4 |
| Rhinolophus ferrumequinum | 48 | 0.01 | 0.7 | 500 | bernoulli | 820 | 4 | 0.05 | 0.5 | lq | 1.02 | 500 | 4 |
| Rhinolophus hipposideros | 9 | 0.1 | 0.7 | 1000 | bernoulli | 380 | 4 | 0.09 | 0.5 | lq | 0.88 | 500 | 4 |
| Rhinolophus mehelyi | 20 | 0.5 | 0.7 | 1000 | bernoulli | 780 | 1 | 0.05 | 0.5 | lq | 2.44 | 500 | 4 |
| Tadarida teniotis | 33 | 0.5 | 0.7 | 1000 | bernoulli | 1000 | 4 | 0.09 | 0.5 | lq | 1.02 | 500 | 4 |
| Vespertilio murinus | 22 | 0.3 | 0.7 | 1000 | bernoulli | 820 | 4 | 0.09 | 0.5 | lq | 4.86 | 500 | 4 |

ANN: Artificial Neural Network; ANN_size: Number of the units in the hidden layer; ANN_decay:Weight decay; ANN_rang: Initial random weights; ANN_maxit: maximum number of iterations; BRT: Boosted regression Tree; BRT_distribution: Name of the used distribution.; BRT_n.trees:Maximum number of grown trees: BRT_interaction.depth: Maximum depth of each tree: BRT_shrinkage.numeric: the shrinkage parameter; BRT_bag.fraction: Random fraction of data used in the tree expansion; Maxent: Maximum Entropy; Maxent_reg: the value of the regularization multiplier used to train the model; Maxent_fc: the feature class combination used to train the model; Maxent_iter: the number of iterations used to train the model; GAM: General Additive Model and GAM_k: folds

**Appendix S7.** Definitions of functional traits used in the study. Traits in red were removed from the analysis because they were highly correlated.

| **Trait** | **Definition** |
| --- | --- |
| Home range | **Spatial trait:** Area routinely used by an individual to satisfy its daily needs. Mean, median min, max and cumulated. Obtained through radio/GPS/stellite tracking studies. |
| Call peak frequency | **Acoustic trait:** Frequency with the greatest amount of energy in a call. Log transformed for analysis. Obtained through acoustic recordings of free flying and hand-released bats. |
| Body mass | **Morphological trait:** Measure the weight in grams. |
| Aspect ratio Index | **Morphological trait:** Calculated as the square of the wingspan divided by the wing area. Log transformed for analysis. Measurements obtained from the literature based on museum specimens or wing tracing/photography of live captured bats. |
| Forearm length | **Morphological trait:** Measured in centimetres. Measurements obtained from the literature or bats measured in the field. |
| Wing load Index | **Morphological trait:** Calculated by body mass divided by wing area (grams/square centimetre). Measurements obtained from the literature based on museum specimens or wing tracing/photography of live captured bats. |
| Estimated Extent Occurence | **Distributional trait:** Area contained within the shortest continuous imaginary boundary which can be drawn to encompass all the known, inferred or projected sites of present occurrence of a taxon, excluding cases of vagrancy. Log transformed for analysis. This trait teases apart species with restricted distribution (i.e. those endemic to one country or adjacent countries) from those only found in southern or northern Europe, and from widely distributed species found across the whole of Europe. Used to represent extinction risk due to restricted distribution. |
| Upper elevation limit | **Distributional trait.** Highest known elevations where the species is found in meters. |
| Foraging habitats | Provided by the IUCN habitat scheme: Arable, pasture, plantation, rural garden, urban, grassland, shrubland, Boreal forest, temperate forest. |
| Dietary diversity | **Dietary trait:** Calculated using Shannon-Wiener diversity index on number of prey orders identified in the diet of the bat species. Log transformed for the analysis. |
| Dietary niche breadth | **Dietary trait:** Calculated using Levin’s standardised index, based  on number of prey orders identified in the diet of the bat species. Log transformed for the analysis. |
| Dietary specialisation | **Dietary trait:** Calculated using the coefficient of variation based on number of prey orders identified in the diet of the bat species. Log transformed for analysis. |
| Thermal index | Thermal preference of a species expressed as the average temperature a species experiences across its geographic range. Geographic range defined based on IUCN range maps. |

**Supplementary Figures**


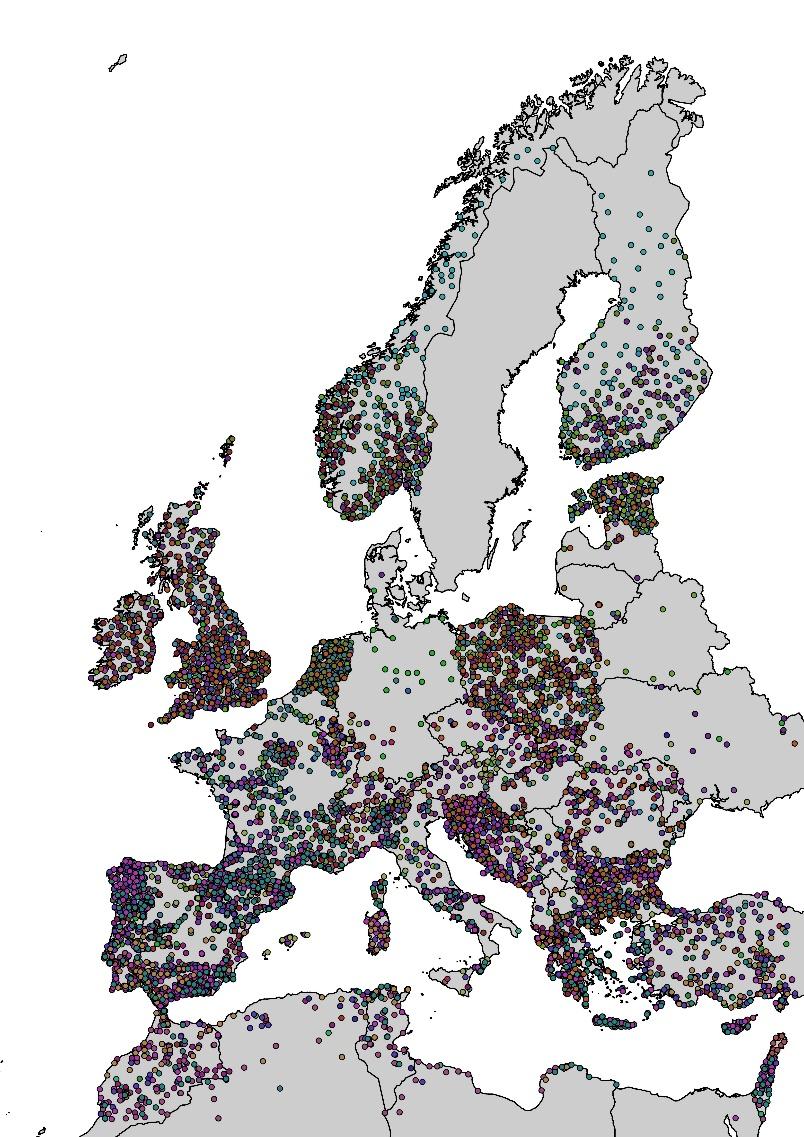

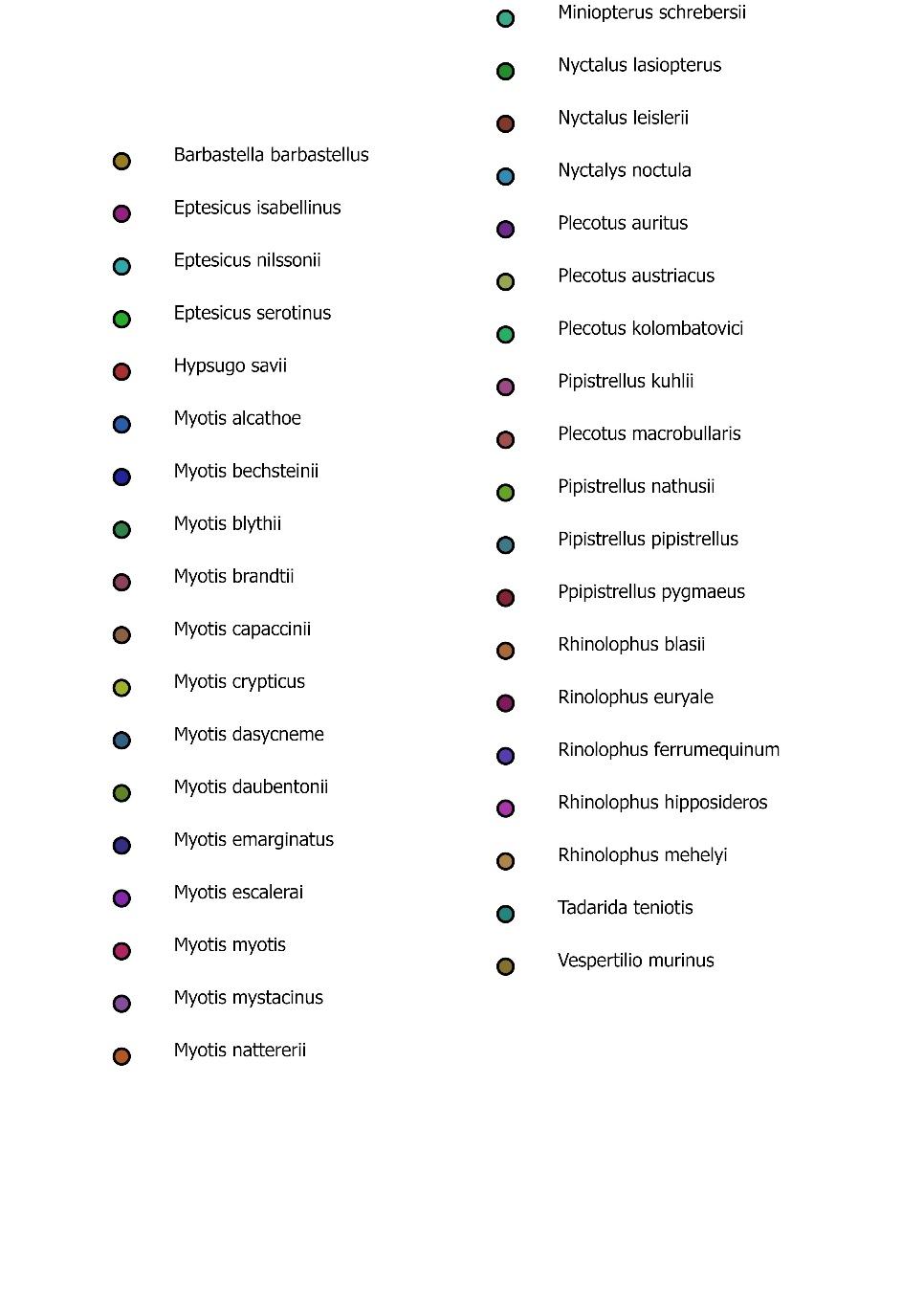

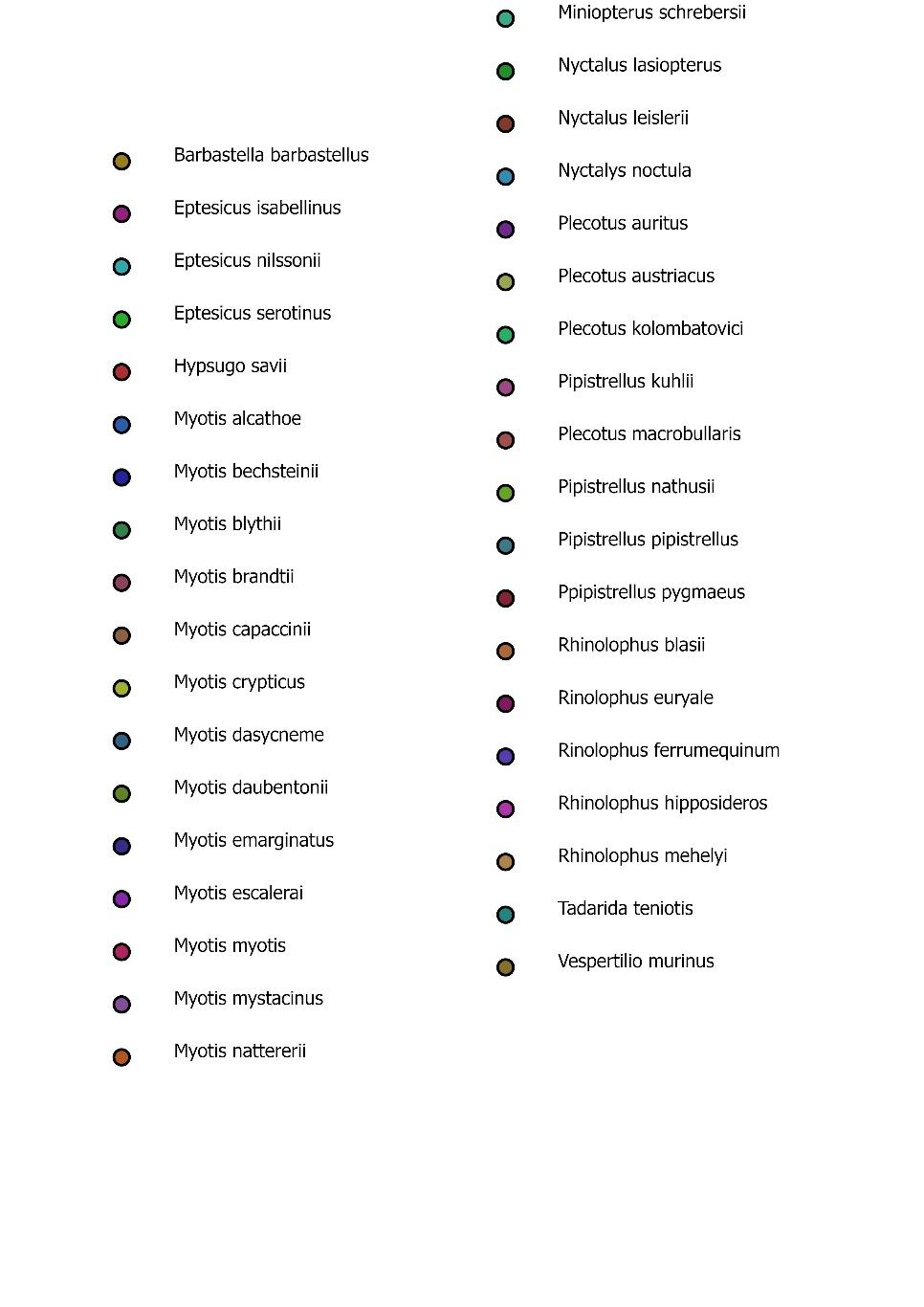

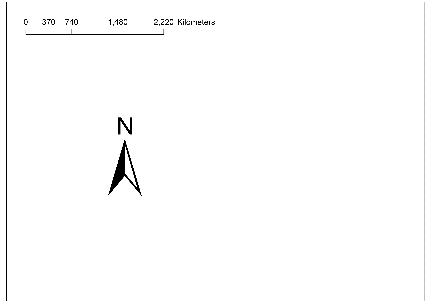

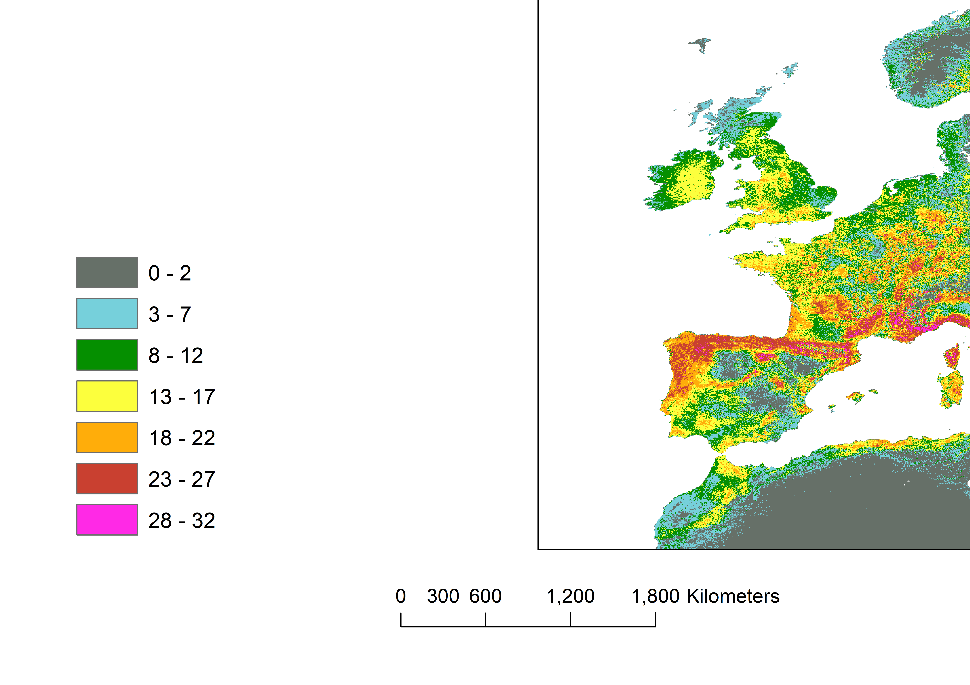


**Appendix S1.** Overview of the study extent and the occurrence records obtained through the European COST ACTION ClimBats network for the 37 bat species across Europe, North Africa and eastern parts of the Mediterranean basin.

**Workflow of specie distribution models Analysis**

**Step 1.1: Content of available datasets**

Step 2.2:

- All variables upscaled to 5km – “raster”
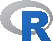

- Remove intercorrelated variables with VIF>10; spearman correlation rho>|0.7|

Step 2.4:

- “SDMTune”
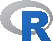

- 70% training and 30% testing data- “biomod2”
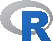

- Binary threshold: random cross-validation
- “blockCV”
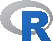


Step 2.7:

- Convert into binary presence/absence maps using TSS scores
- Future- mean 3 GCMs
- “biomod2”
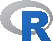

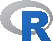


Step 2.5:

- Averaging based on AUC and TSS
- excluding models AUC<0.75
- “biomod2”
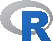

- Unpublished OR from bat experts “Climbats” COST ACTION NETWORK (CA18107)
- EUROBAT dataset
- N. Africa OR from literature

Cleaning and spatial filtering

**Step 2.7: Generate Present /future maps; range shifts and overlap.**

**Step 2.3: Model calibration & evaluation**

Parameterization of each species for each algorithm

4 algorithms

ANN, GAM, GBM, MaxEnt

**Step 2.5: Ensemble models means.**

Performance evaluation: ROC, TSS & block cross-validation

**Step 2.1: Species Distribution Modelling (SDM)**

Environmental data- Current

- 6 Bioclimatic variables at 30arc-second resolution – CHELSA-Climate
- 2 land cover variables at 300m resolution – Globio4 map
- 1 topographic at 30m – Copernicus Elevation

Environmental data- Future

- 6 Bioclimatic variables projected for 2041-2060.
- RCP 4.5 & RCP 8.5
- 3 GCM: HadGEM2, IPSL-CM5A and MPI-ESM


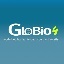

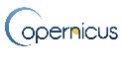


**Step 2.6: Project to the future** 2041-2060 (RCP4.5 & 8.5); 3GCMs

Step 1.2:

- Remove duplicate OR
- Thinned OR to 40km

“spThin”

Tab. S2

Fig. S1

Tab. 1

Tab. S3

Tab. S4

Fig.1

Fig. S4

Step 3.2:

- Binary maps added to produce species richness map present/future

Step 3.3:

- Community turnover and nestedness between present/future
- Jaccard dissimilarity index
- “betapart”
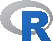


Step 3.4:

- 10 traits – EurobatTrait
- Filled gap with imputation -missForest
- Functional dispersion (Fdis) & RaoQ- “FD”
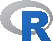

- Community Weighted Mean (CWM) – “FD”
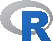


**Step 3.2 Species richness**

**Step 3.1: Species richness, composition and functional diversity**

**Step 3.3 Community composition b-diversity**

**Step 3.4 Functional diversity & composition**


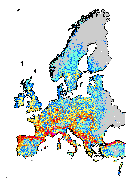

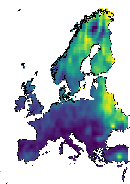

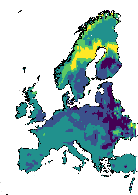


Fig. 2

Fig. 3

Fig. 3

Fig. 4

Tab. S5

**Figure S2.** Methodological framework applied to predict the impact of climate change on range suitability, species richness, community composition and functional diversity for 37 European bat species. The blue boxes are the results of the analyses.


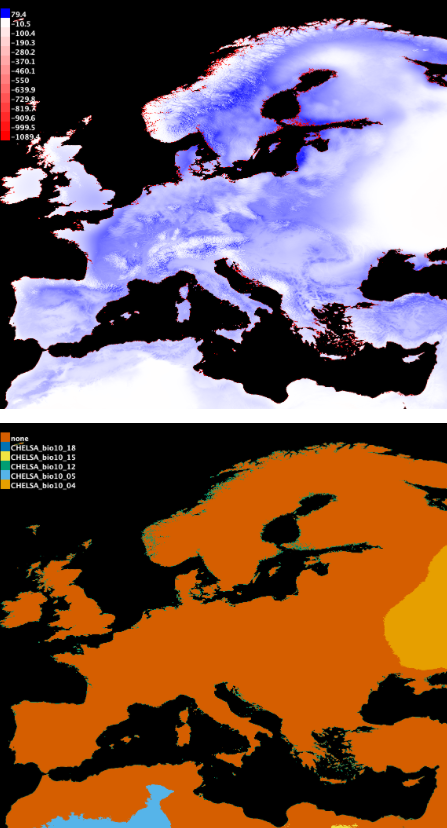


**Appendix S6.** Multivariate Environmental Similarity Surfaces (MESS) plots. a) MESS output map showing areas across Europe and North Africa where environmental variables are outside the range present in the training data. Red represents areas where one or more variables are outside their training range. b) MESS output map showing the most dissimilar variables outside their training range. Orange colours show that none of the variables are outside their training range.


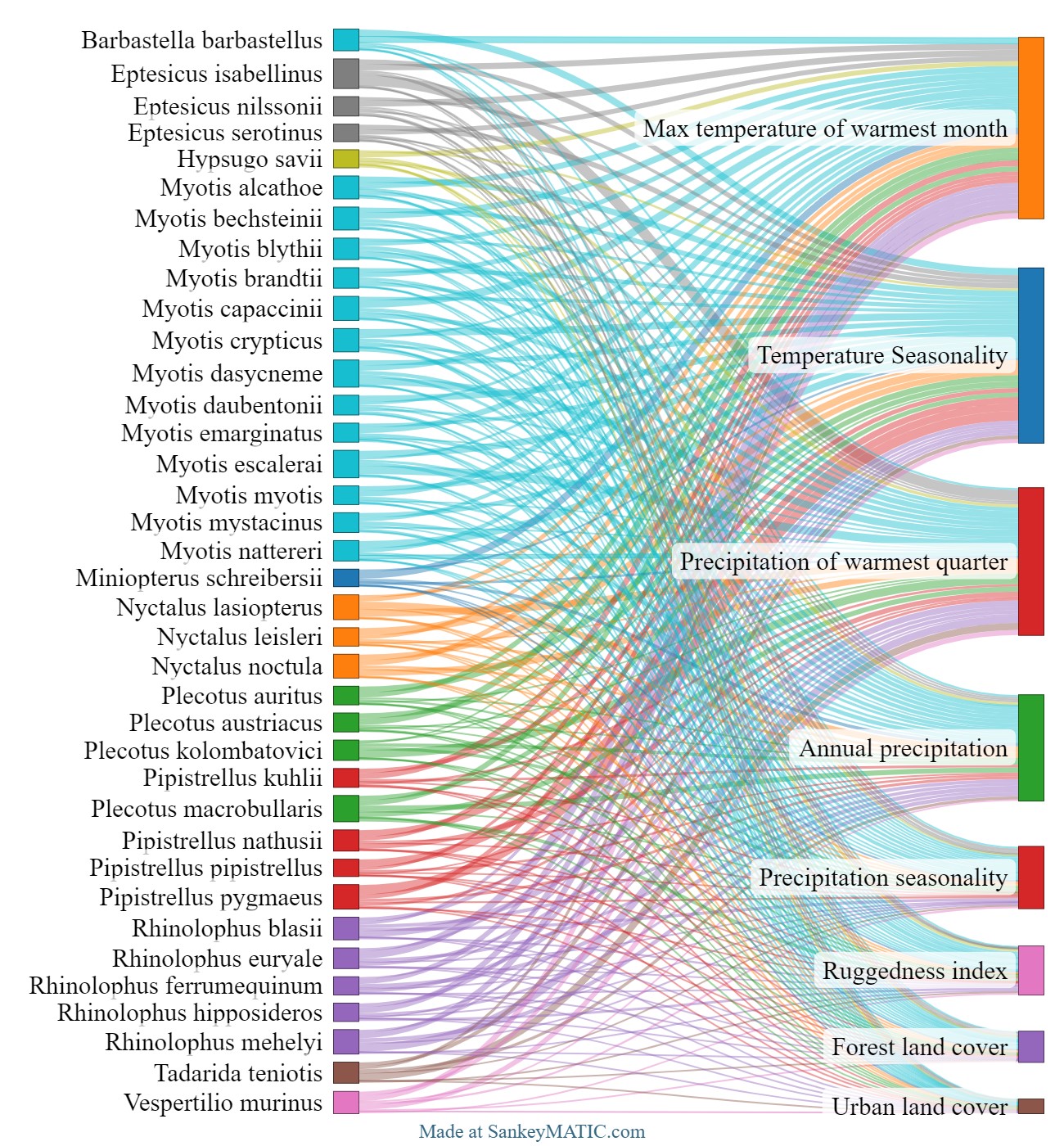


**Appendix S8.** Sankey diagram showing the contribution of the environmental variables to the species distribution models for each bat species. The width of the lines represents the strength or weight of the contribution of each variable to the model for each species.

**Future RCP 8.5**

**Future RCP 4.5**

**Present**


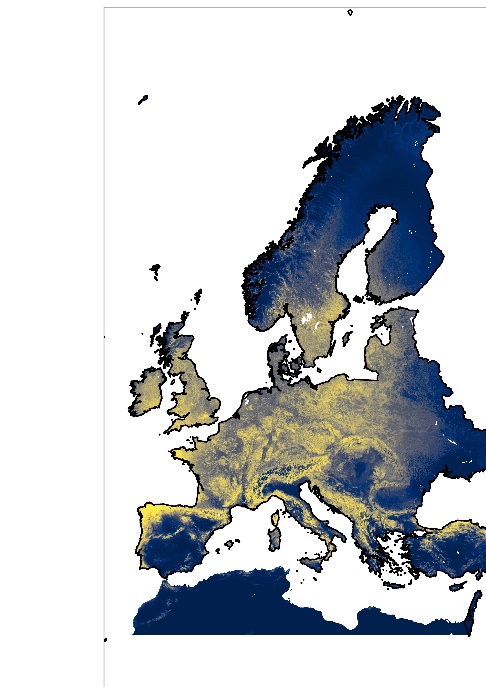

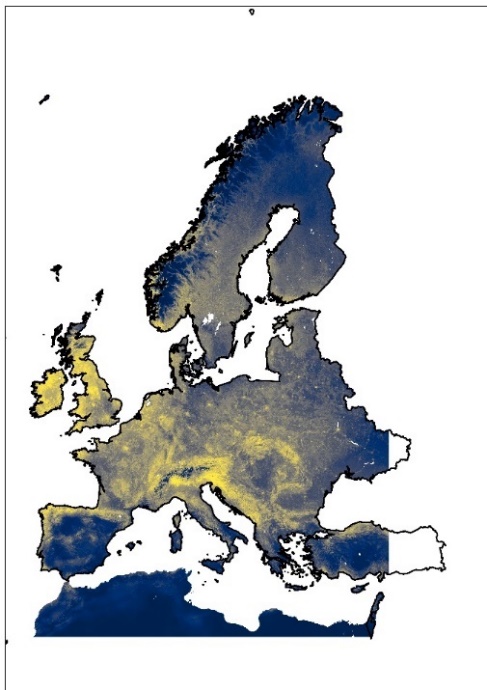

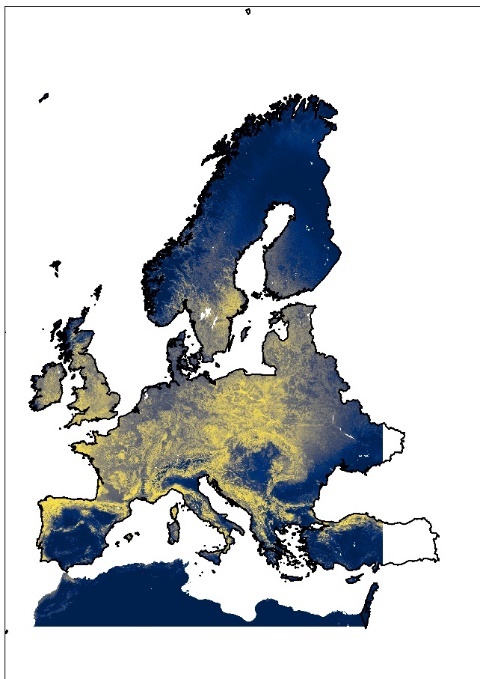


*Barbastella barbastellus*


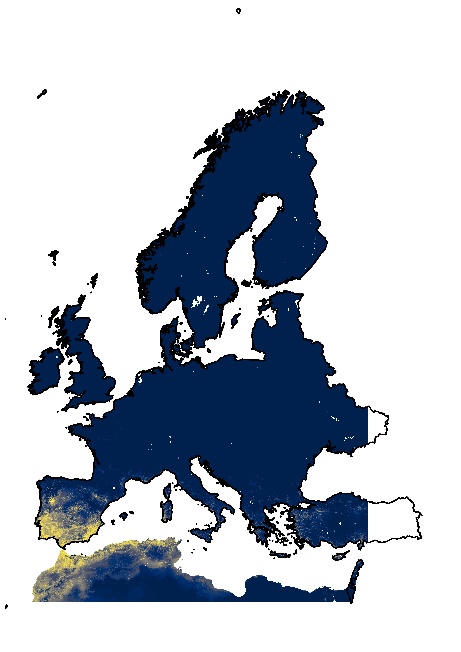

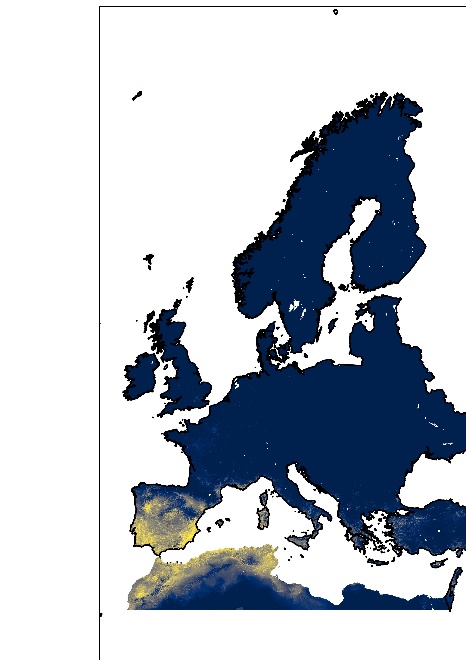

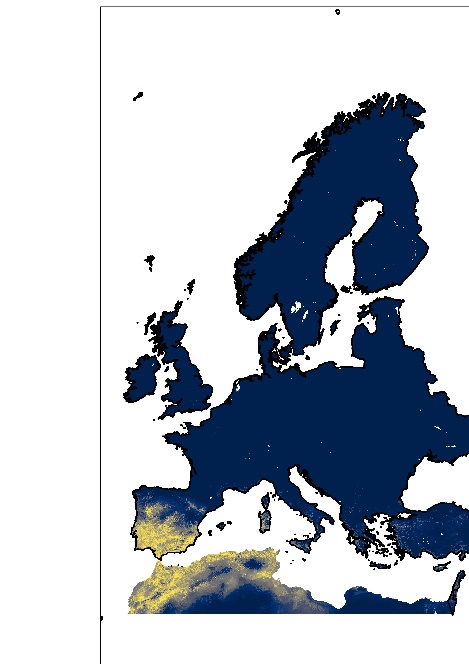


*Eptesicus isabellinus*


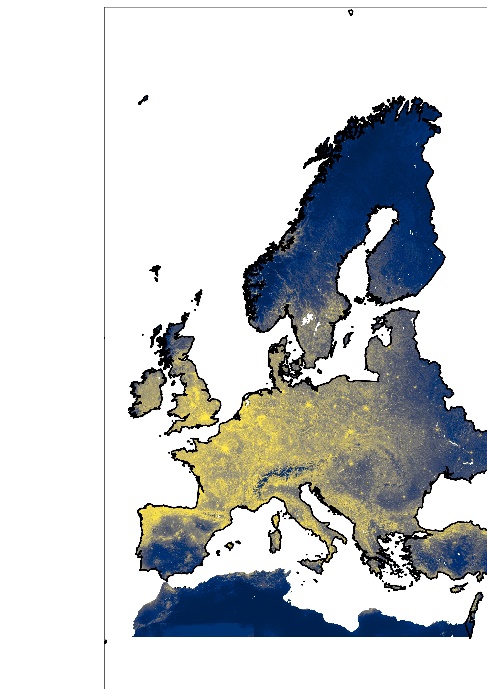

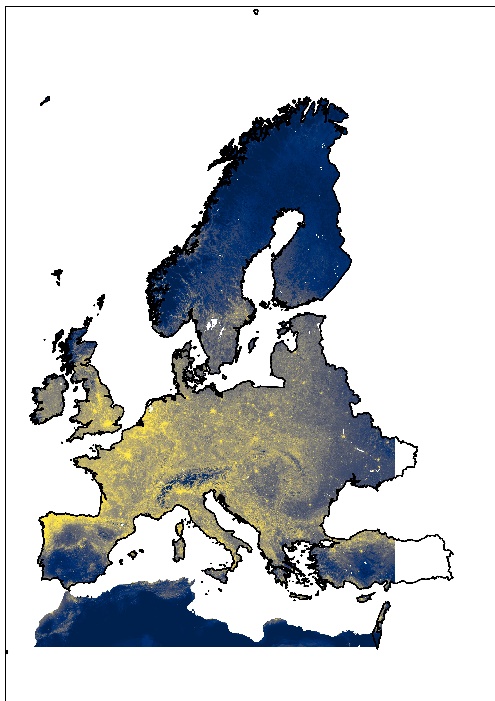

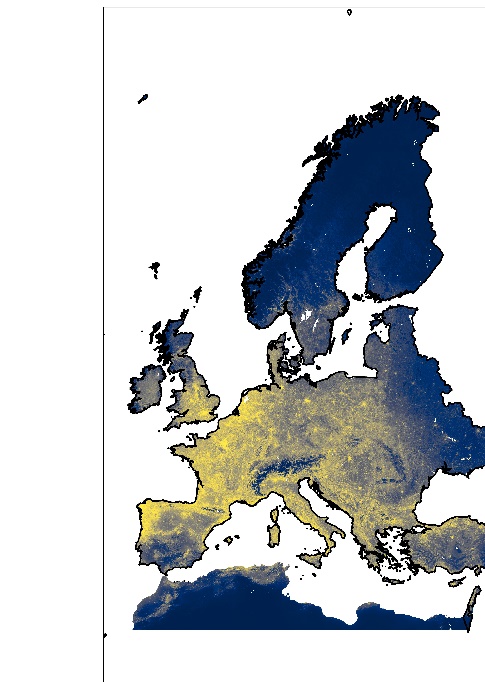


*Eptesicus serotinous*


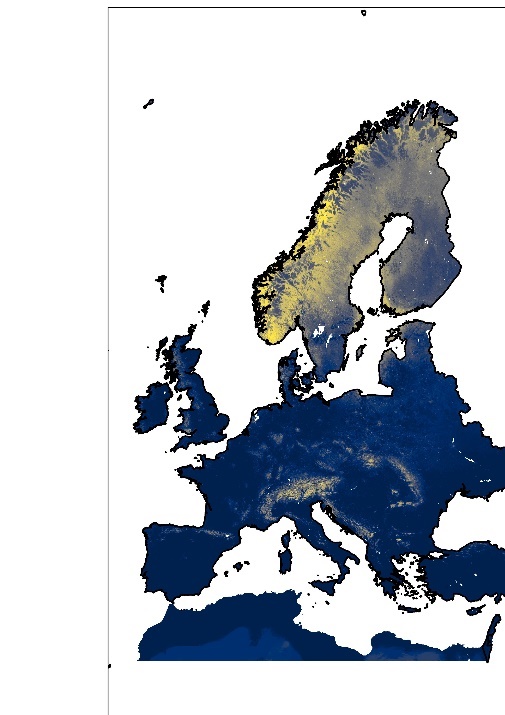

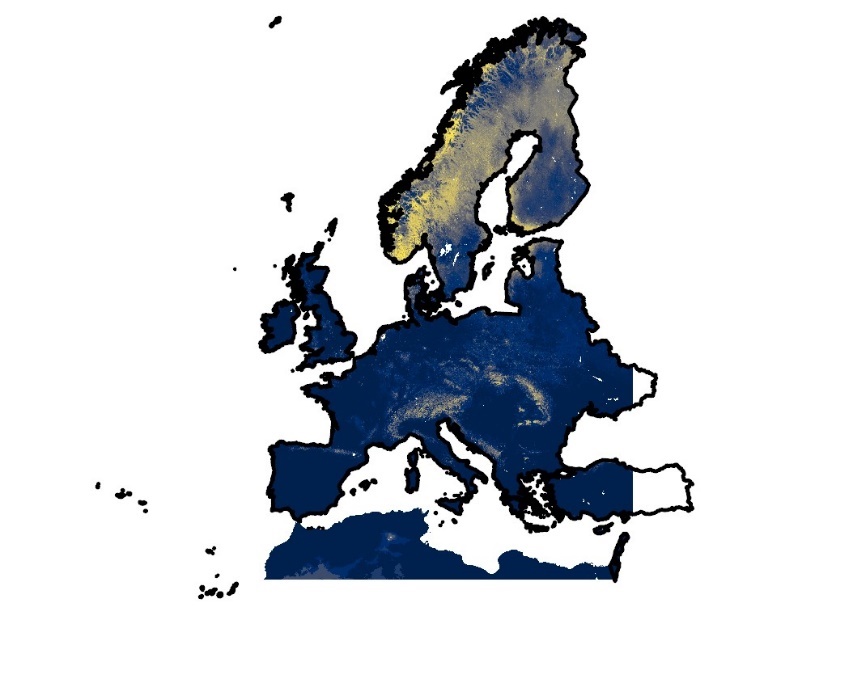

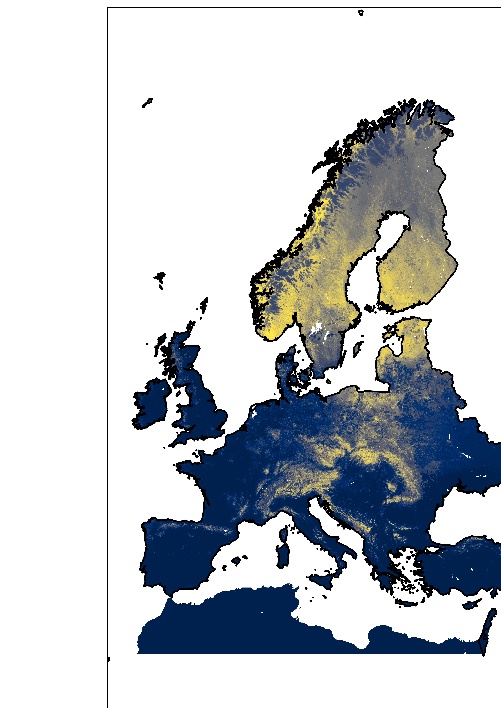


*Eptesicus nilsoni*


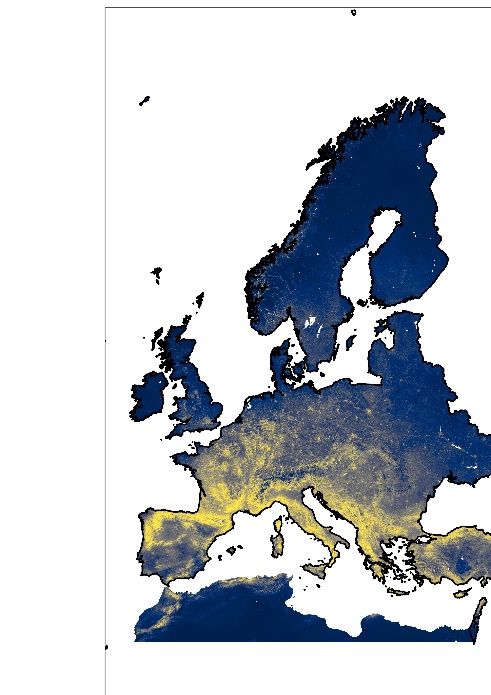

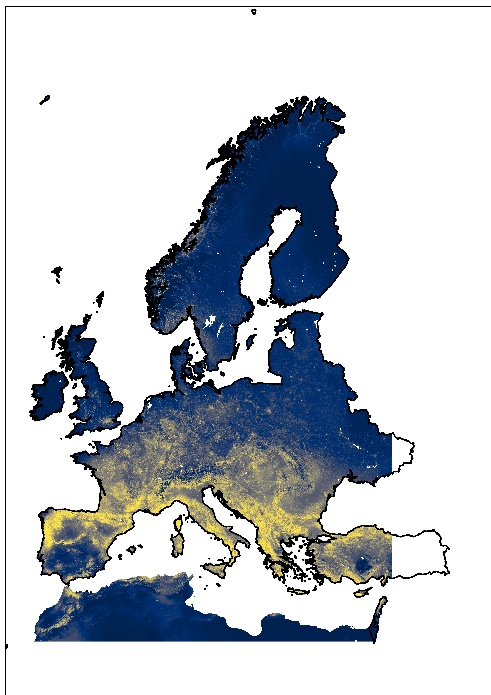

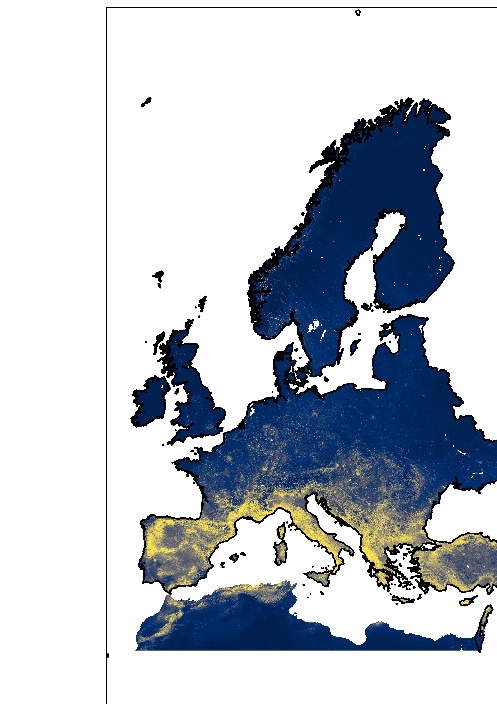


*Hypsugo savii*


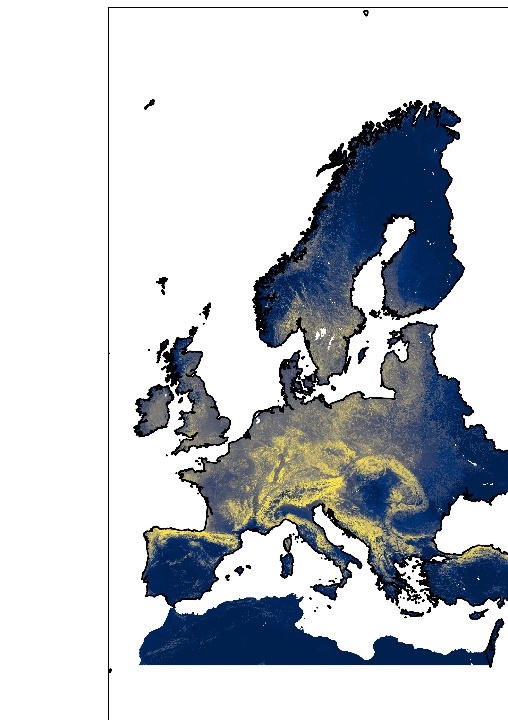

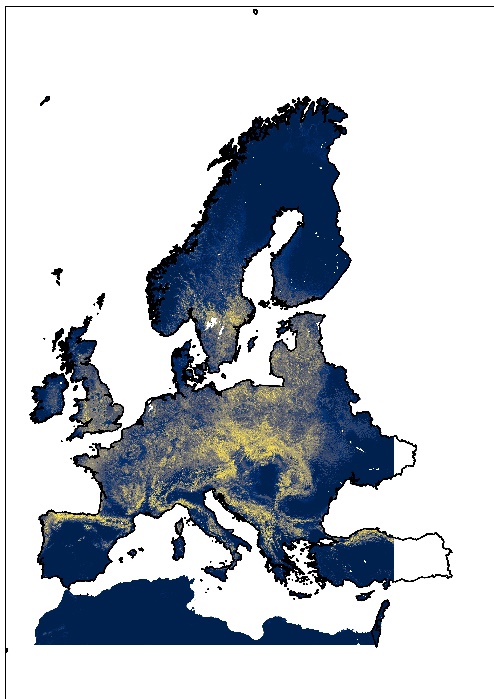

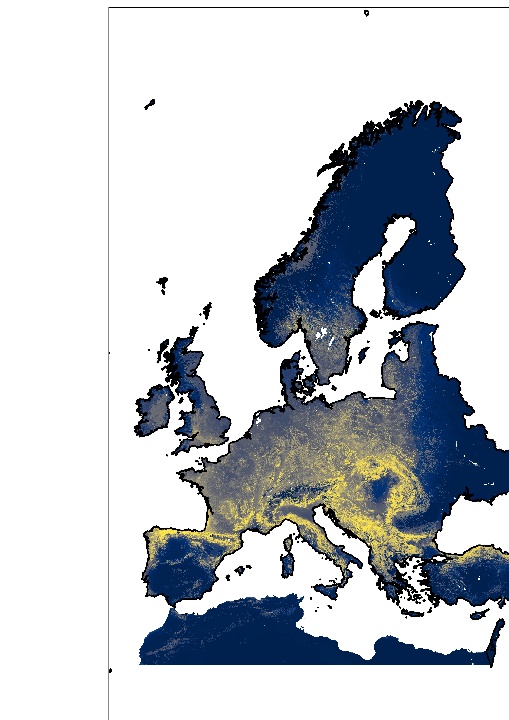


*Myotis alcathoe*


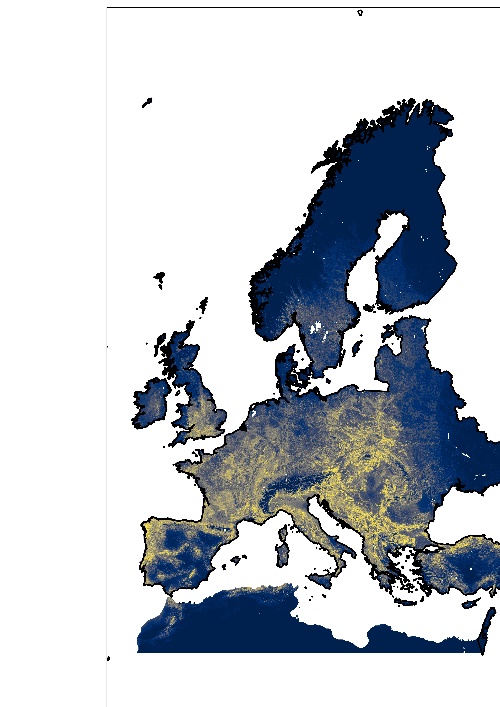


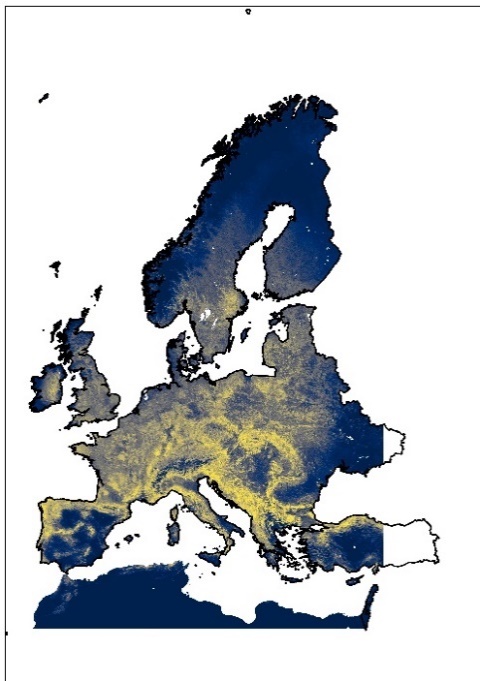

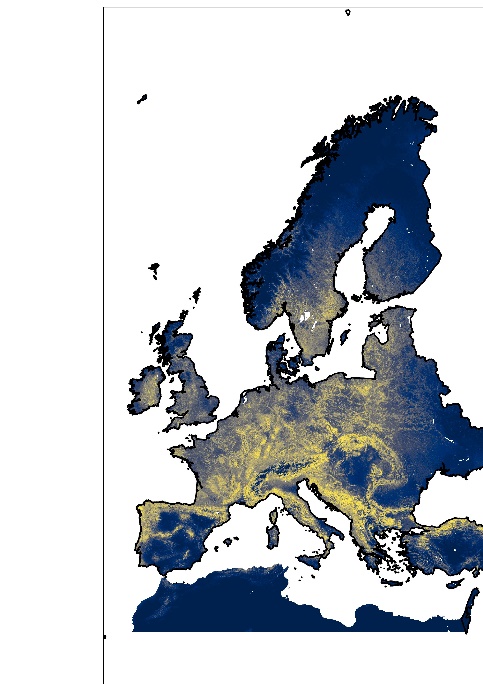


*Myotis bechsteini*


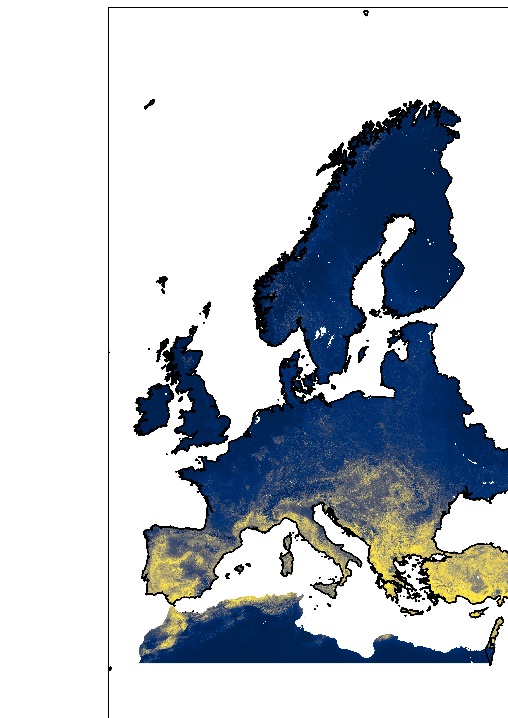

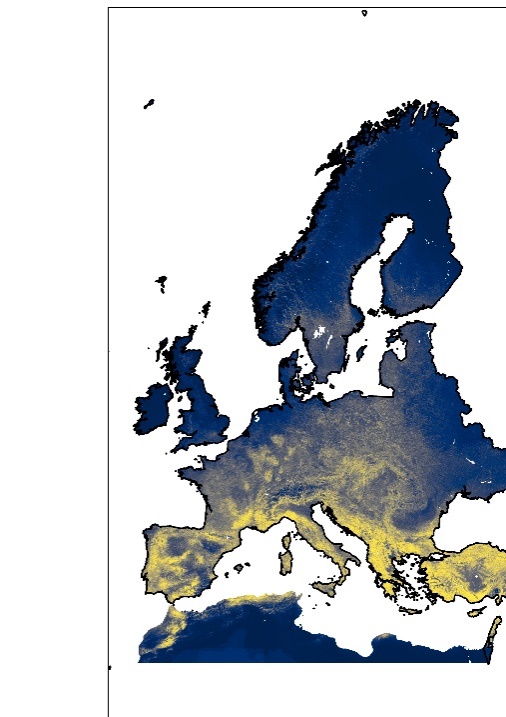

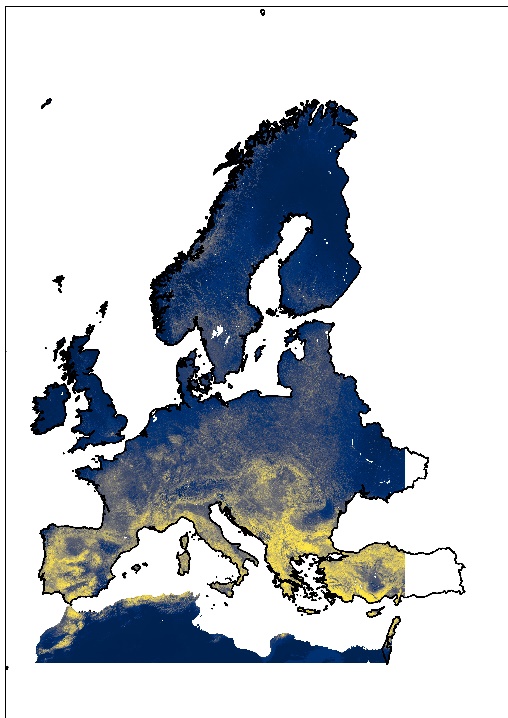


*Myotis blythii*


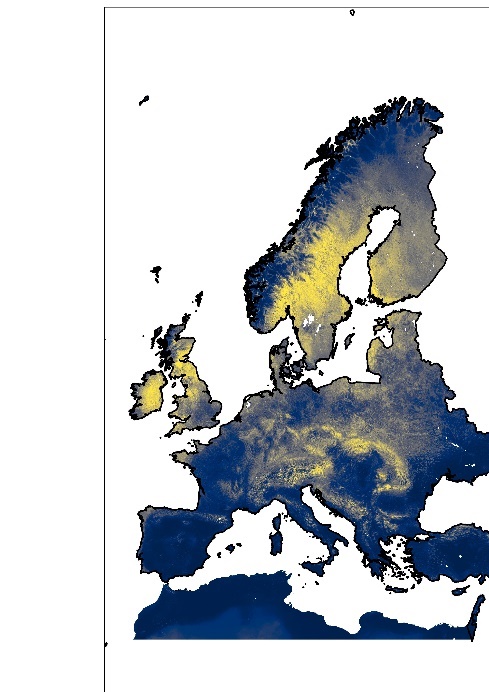

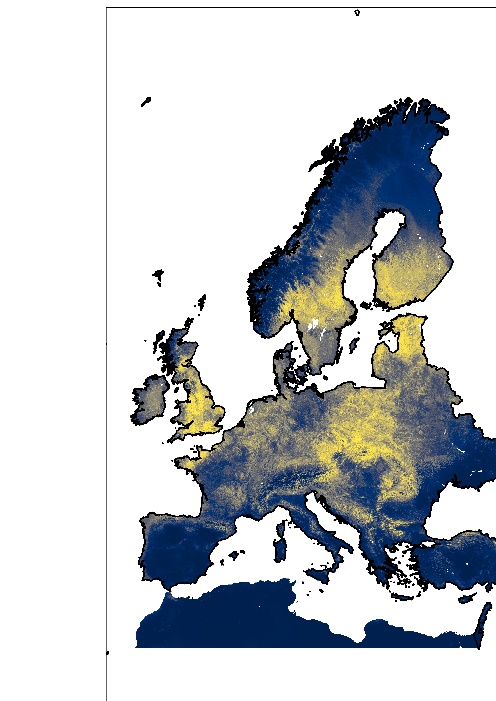

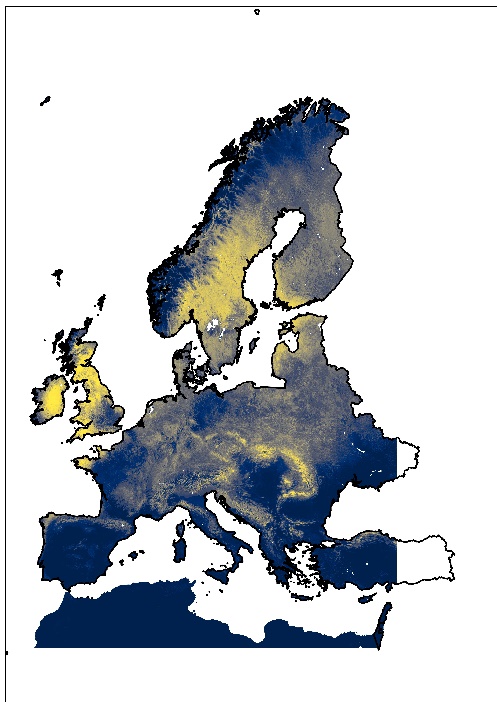


*Myotis brandtii*


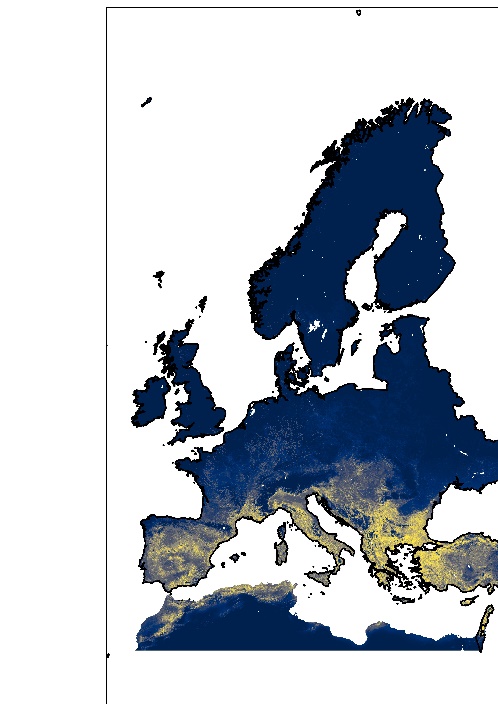


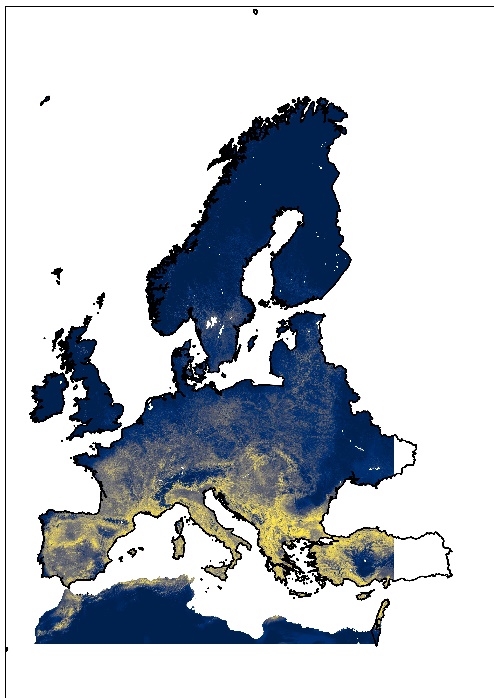

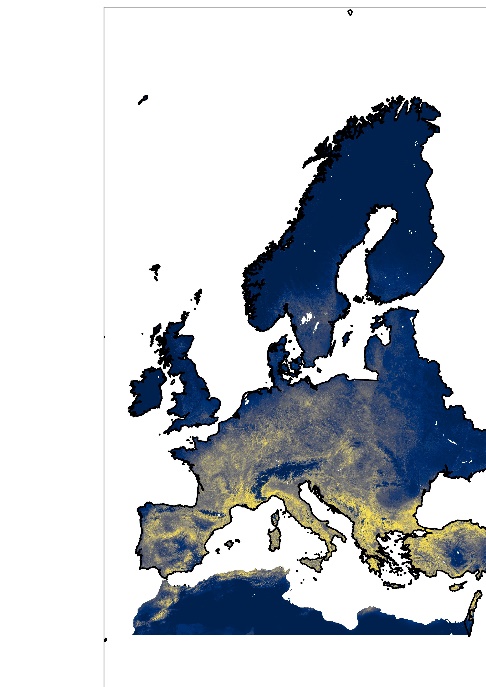


*Myotis capaccinii*


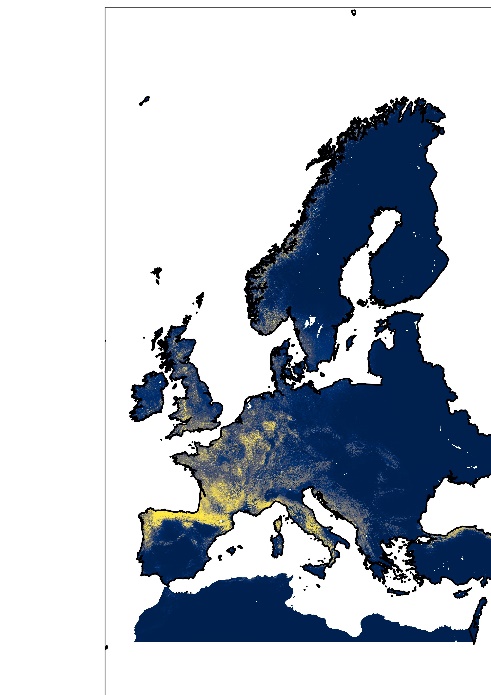

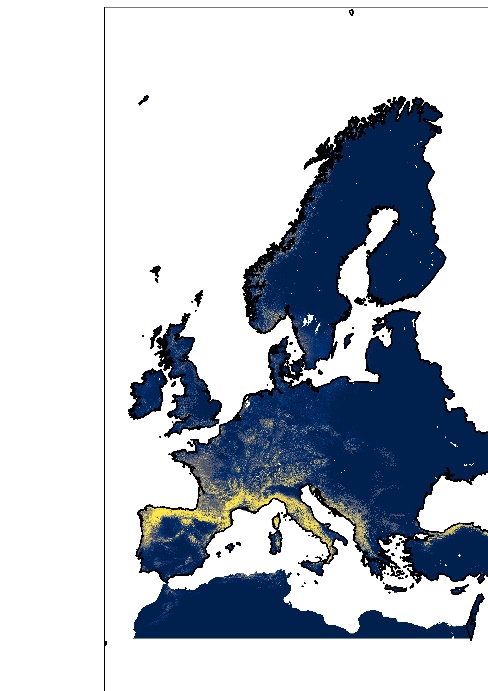

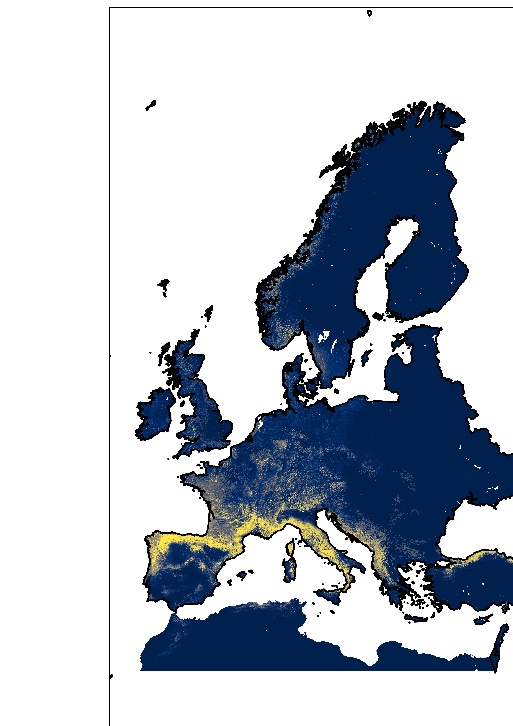


*Myotis crypticus*


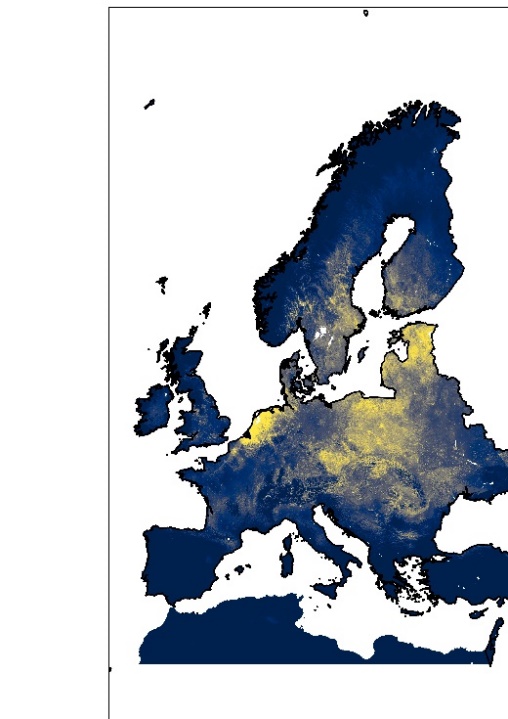

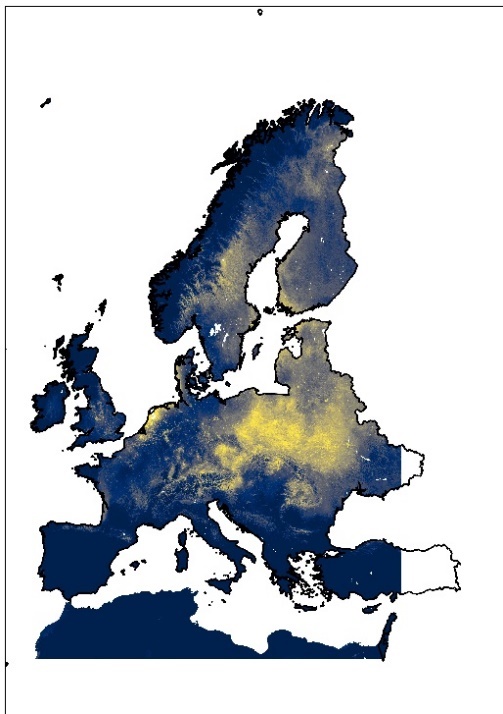

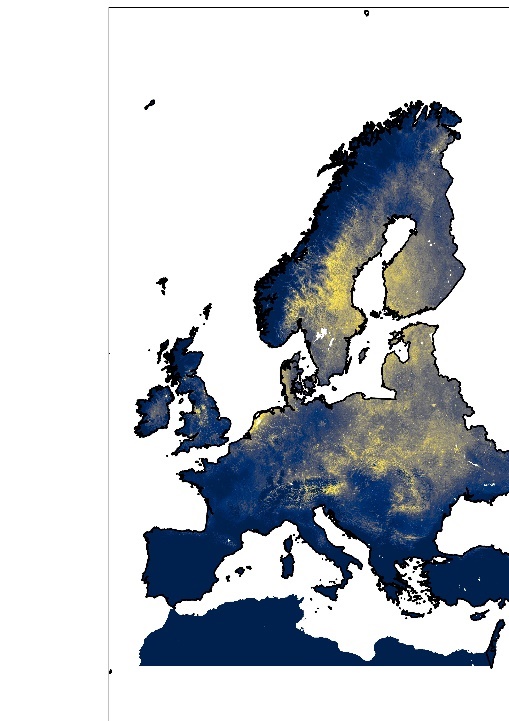


*Myotis dasycneme*


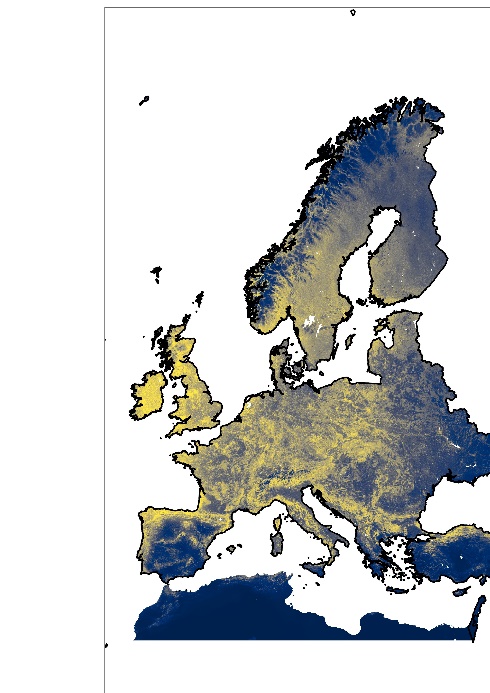

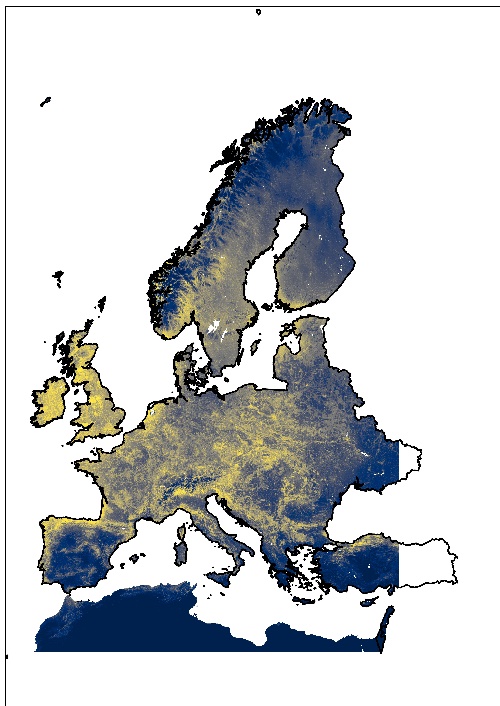

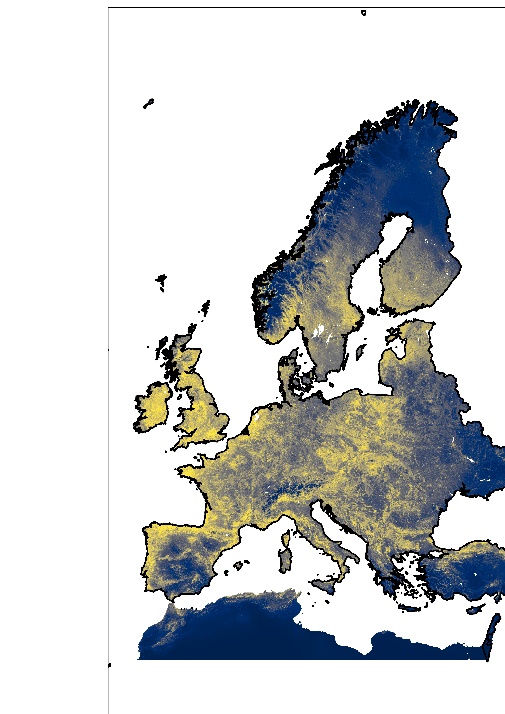


*Myotis daubentonii*

1`
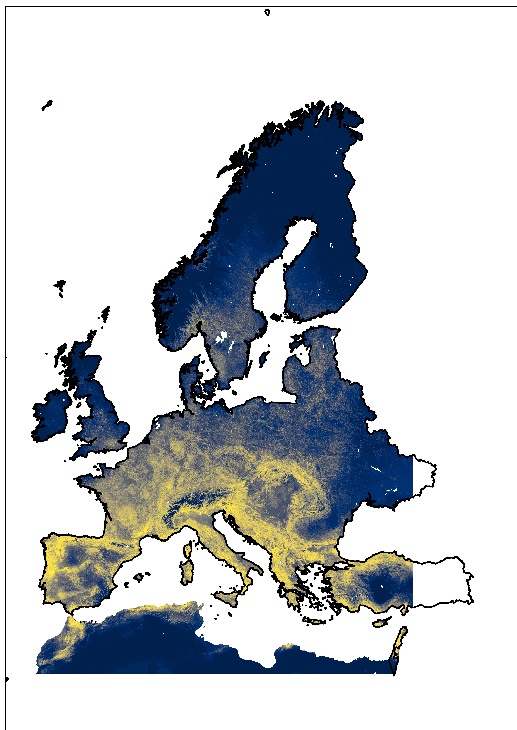

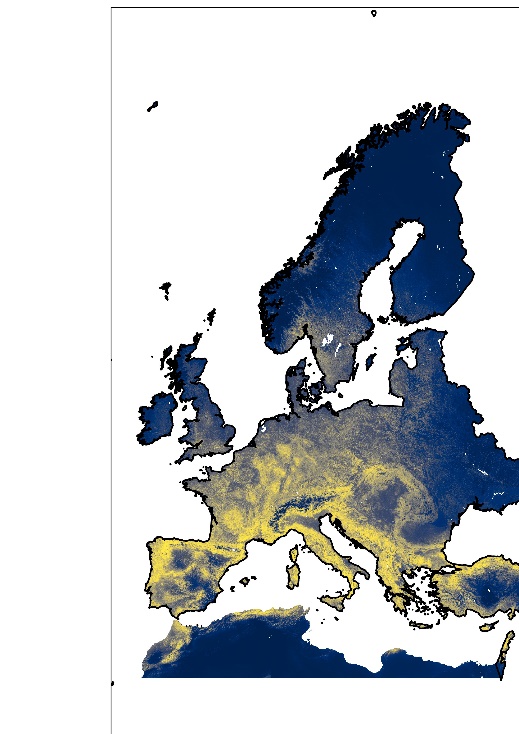

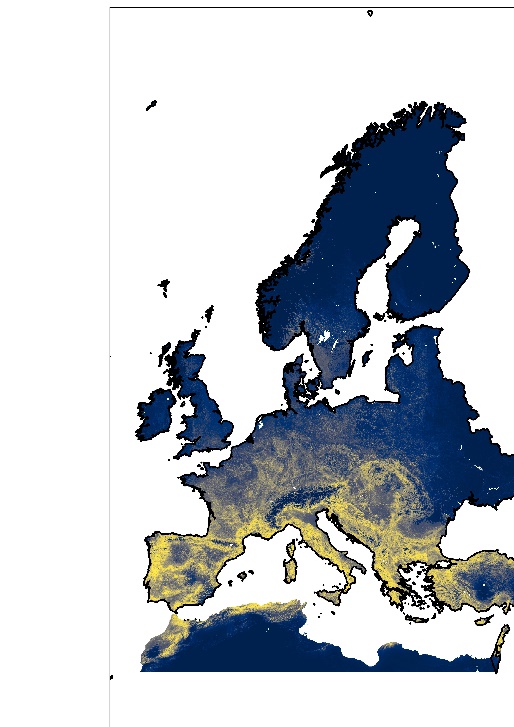


*Myotis emarginatus*


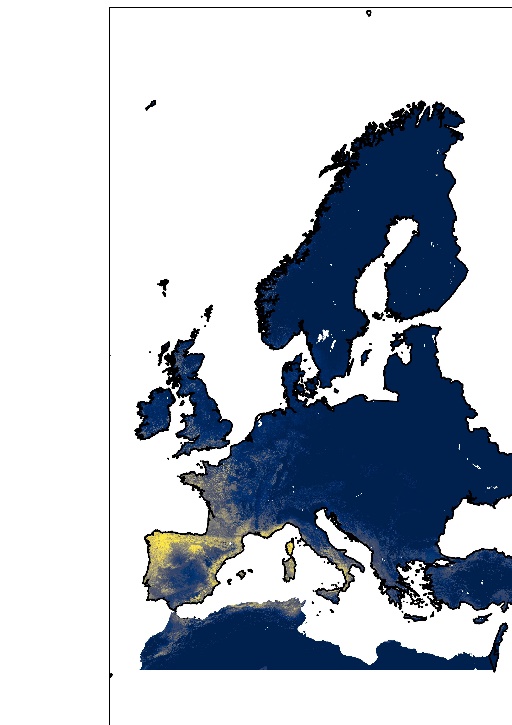

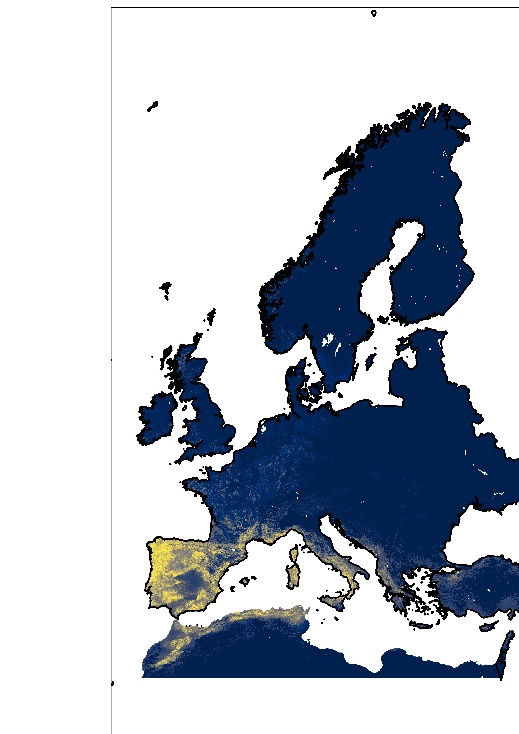

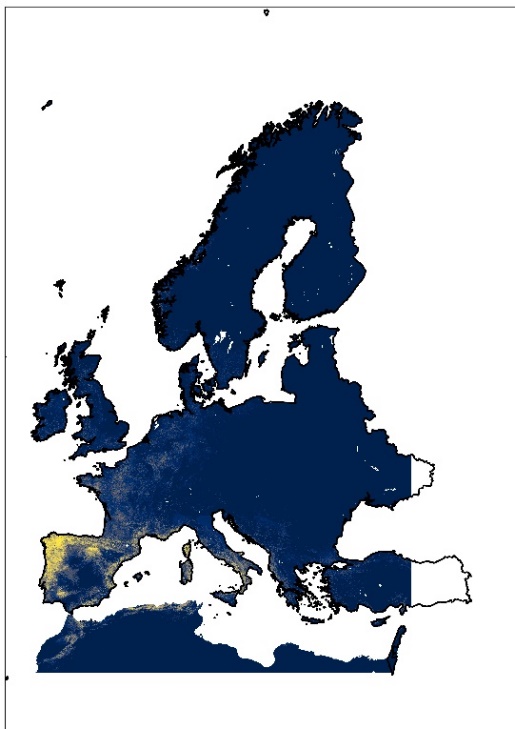


*Myotis escalerai*


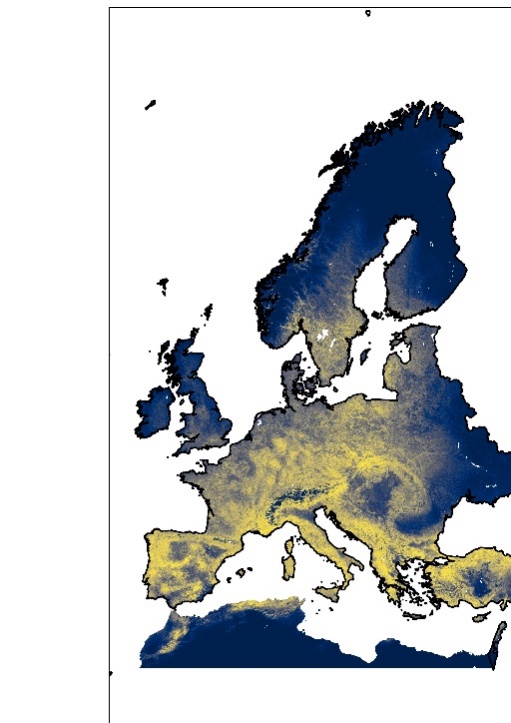

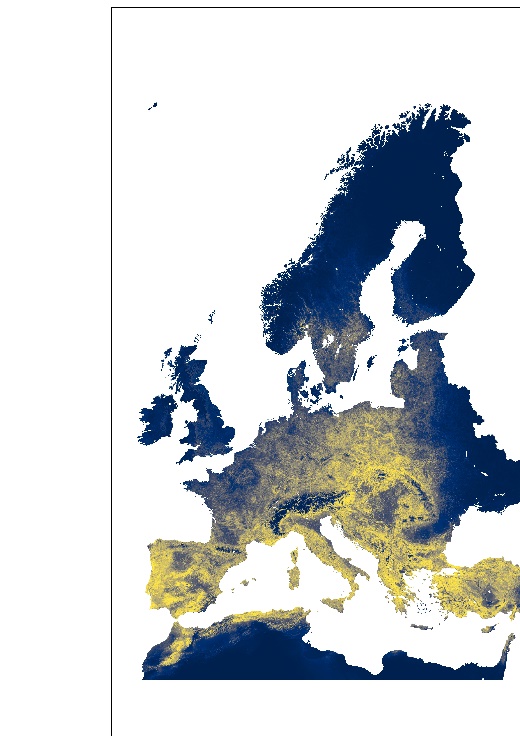

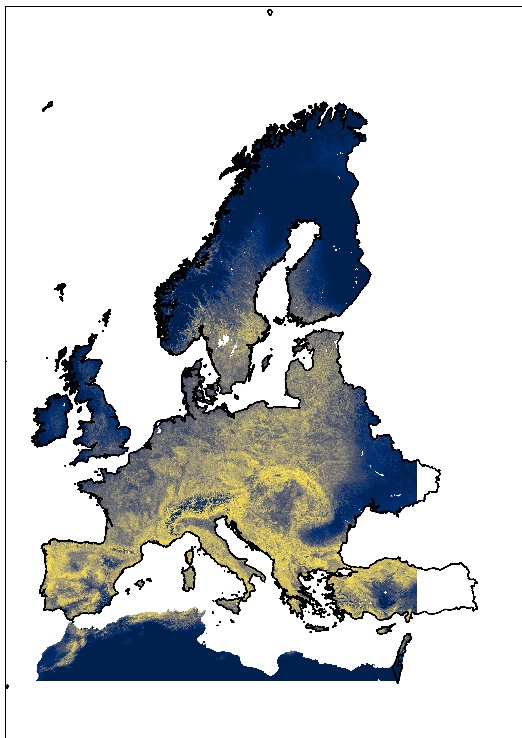


*Myotis myotis*


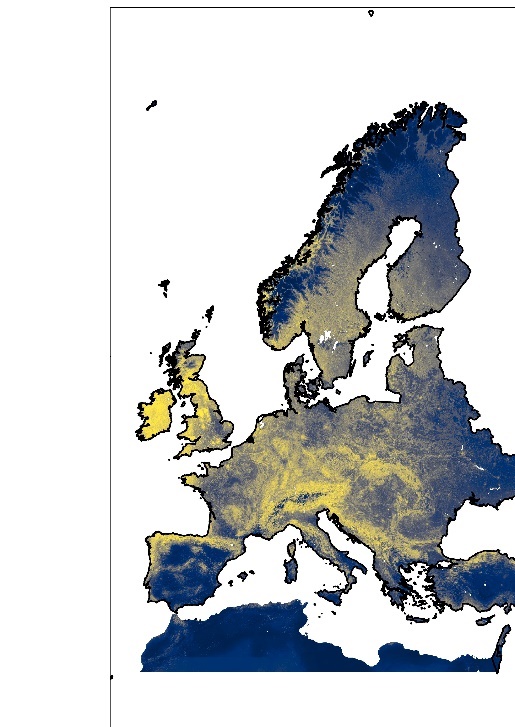

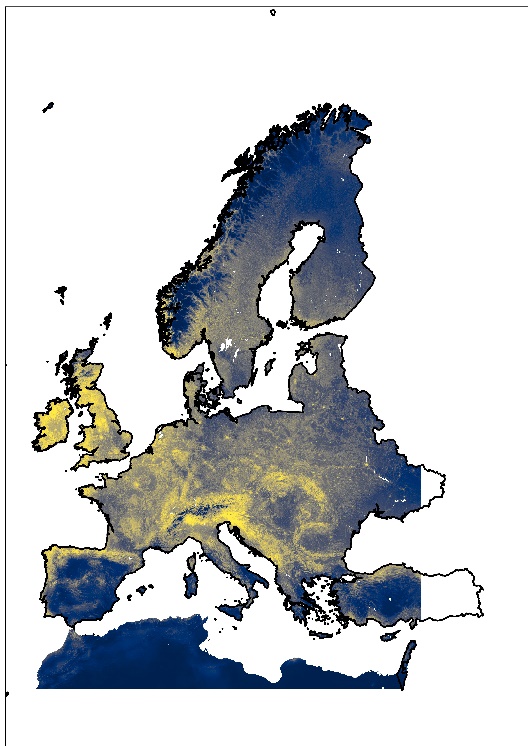

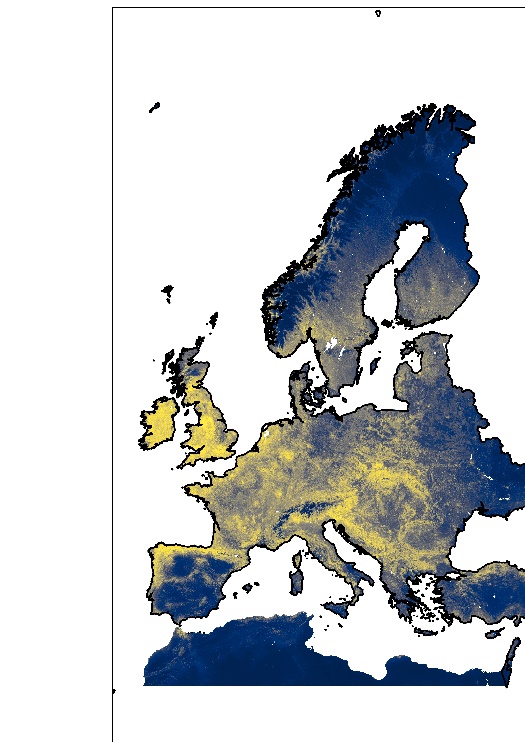


*Myotis mystacinus*


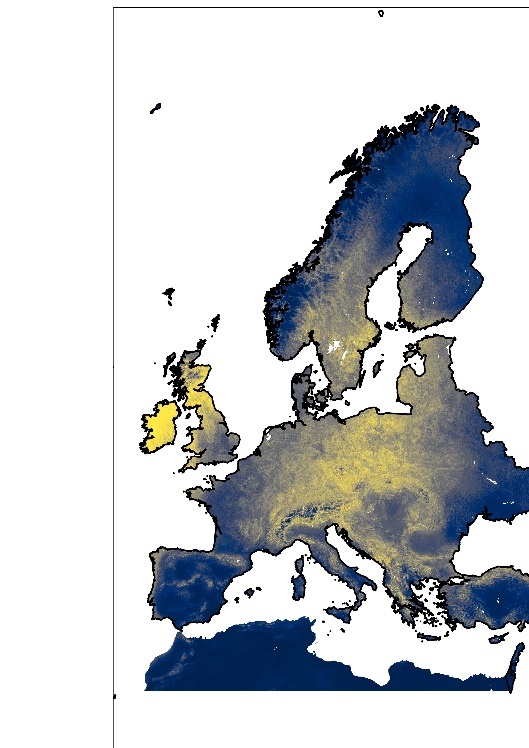

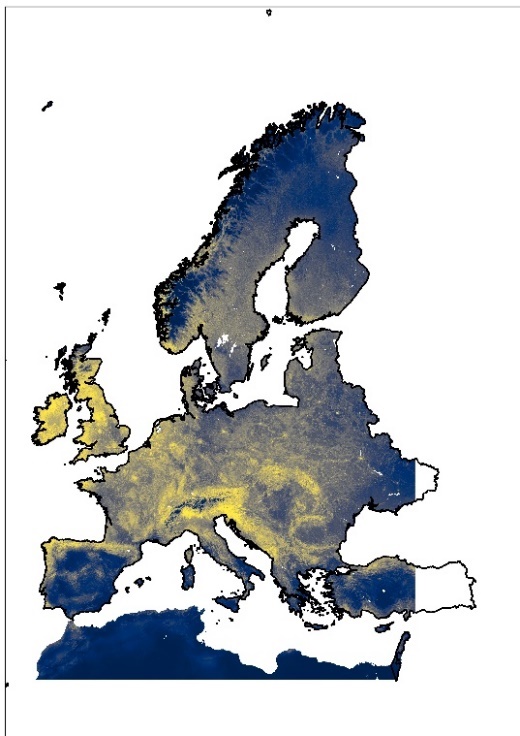

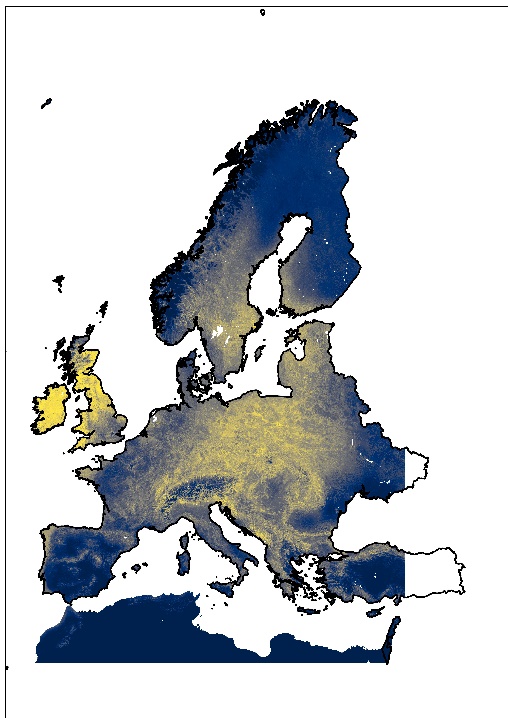


*Myotis nattererii*


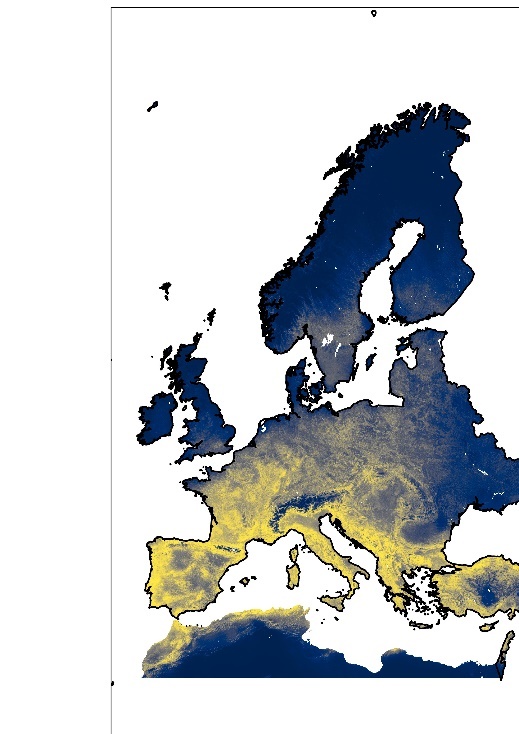

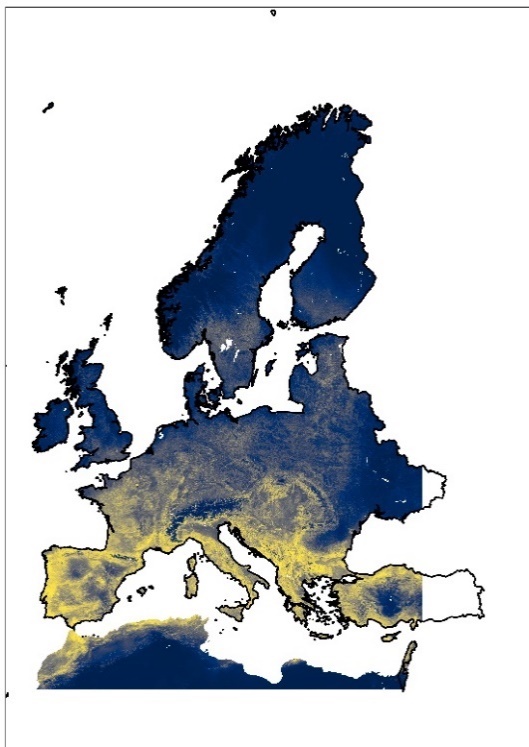

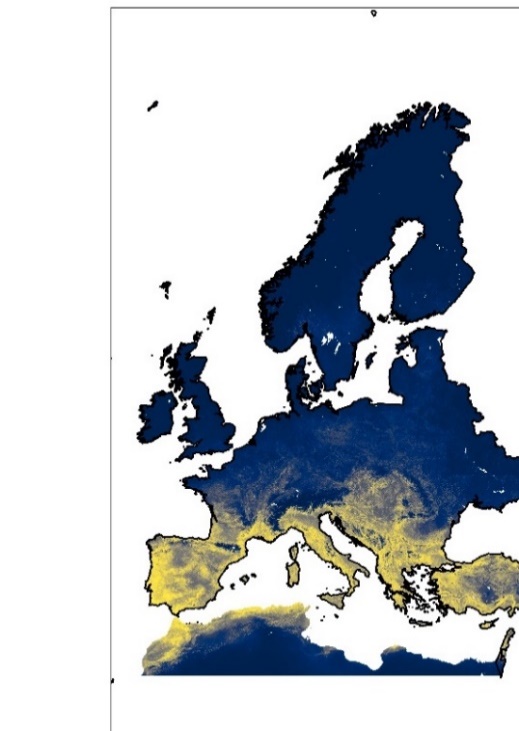


*Miniopterus schrebersi*


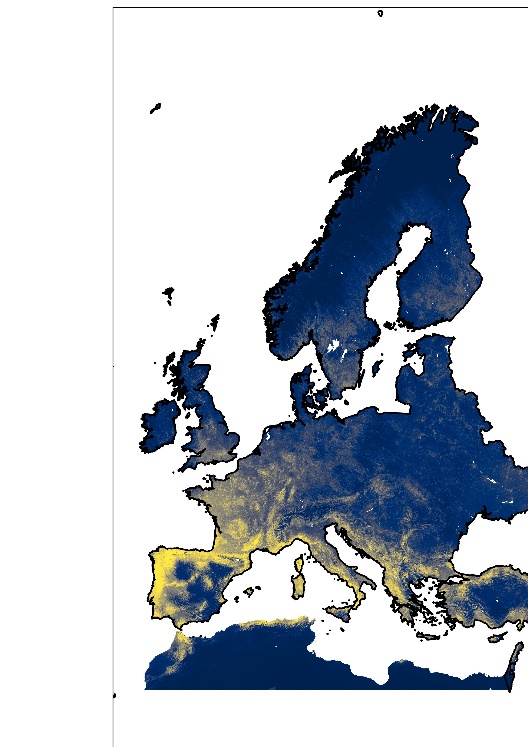

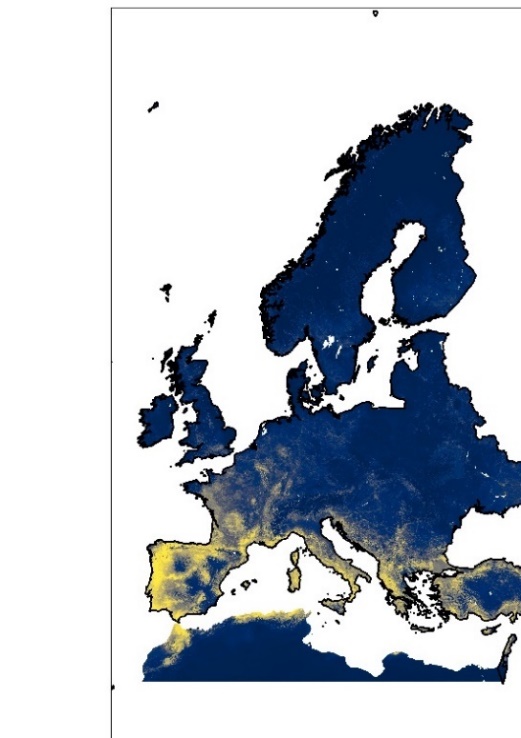

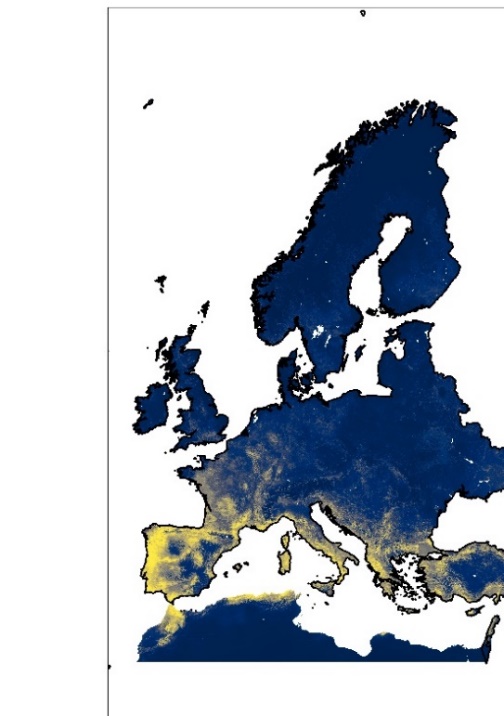


*Nyctalus lasiopterus*


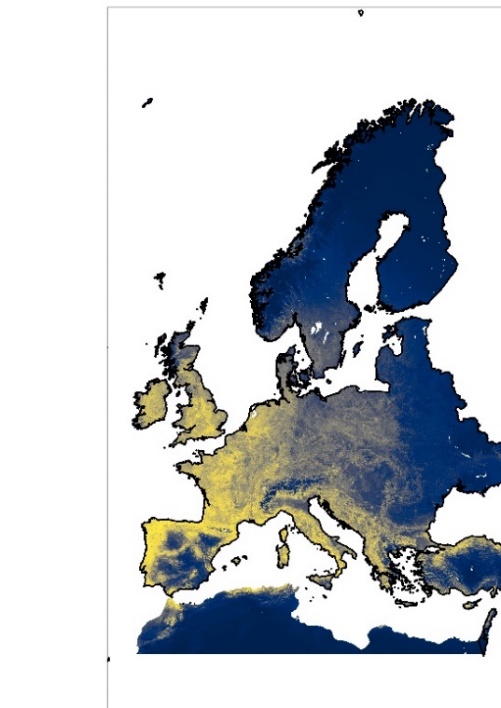

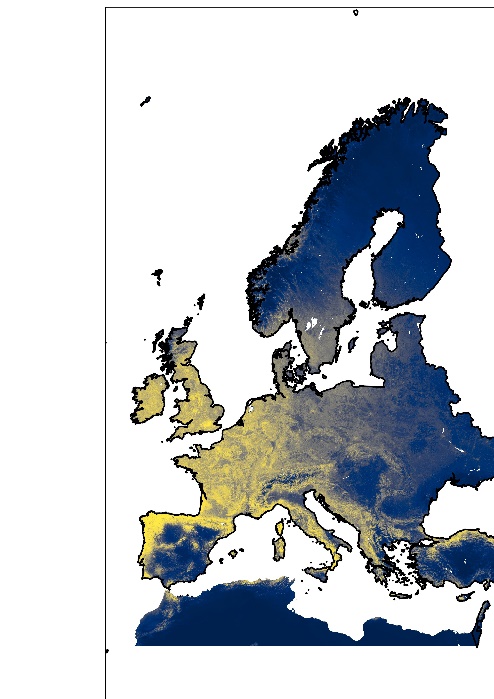

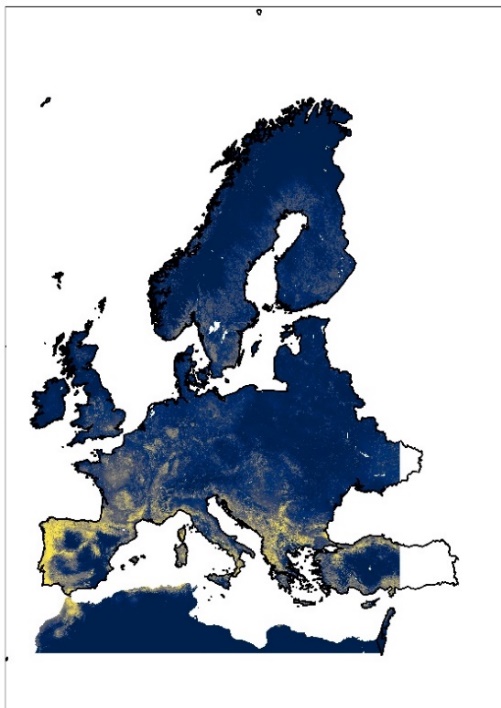


*Nyctalus leisleri*


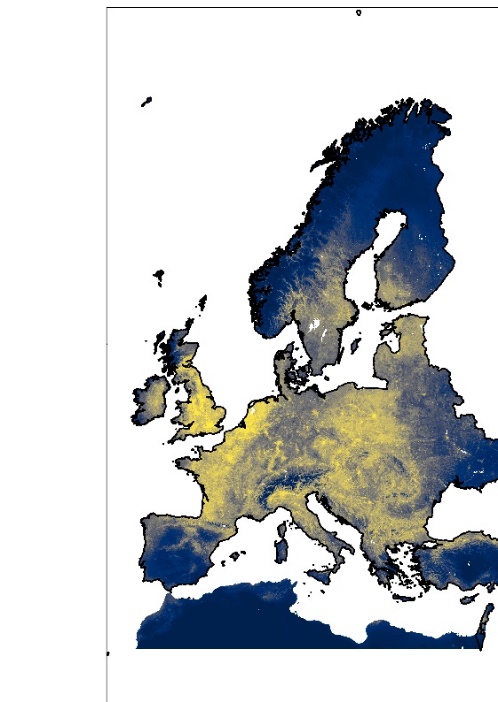

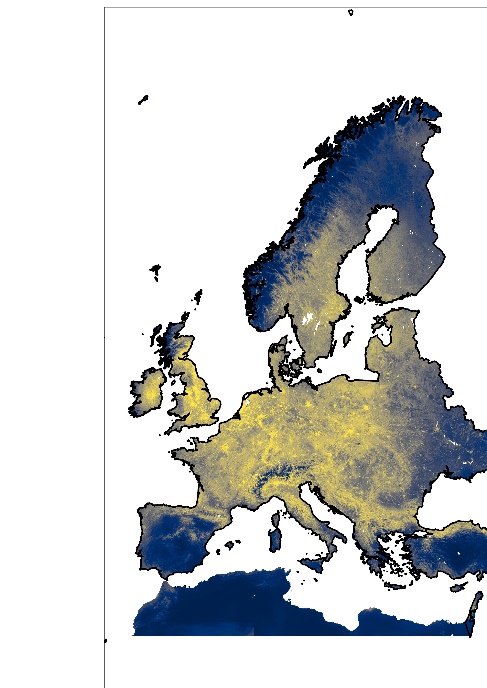

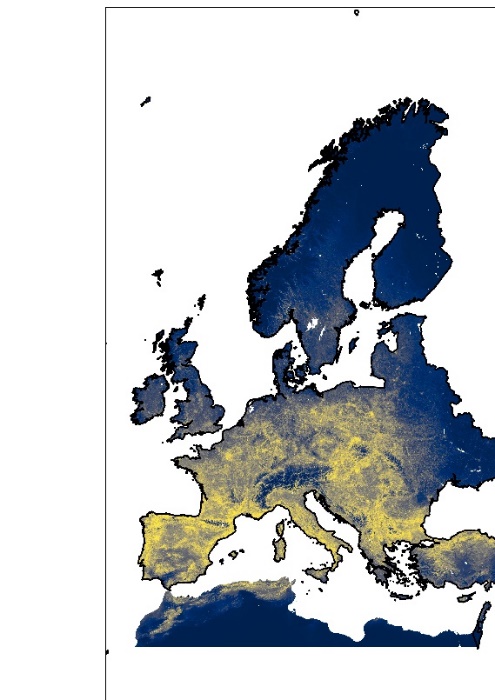

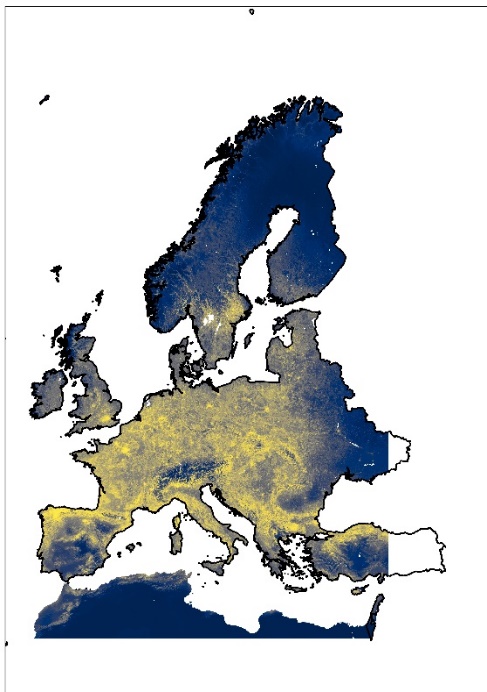

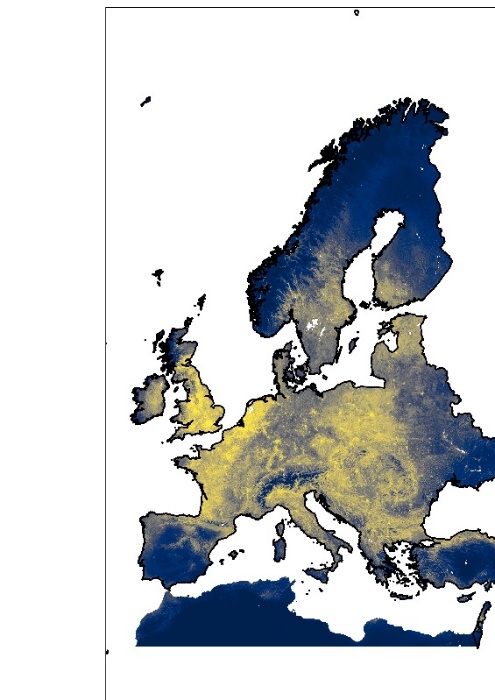


*Nyctalus noctula*


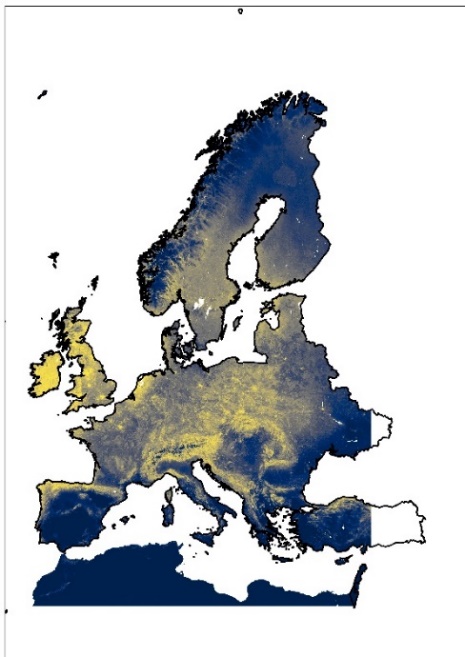

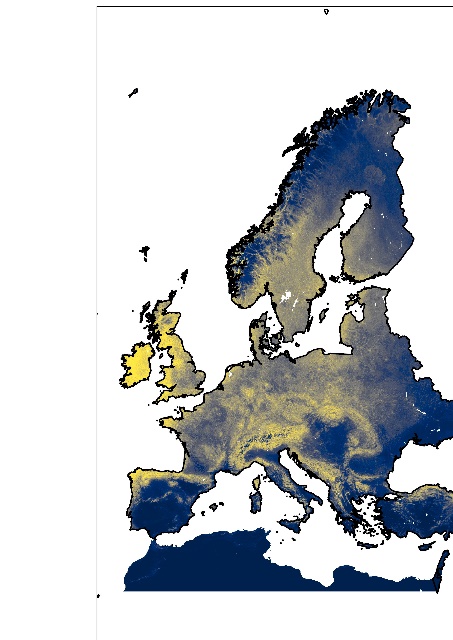

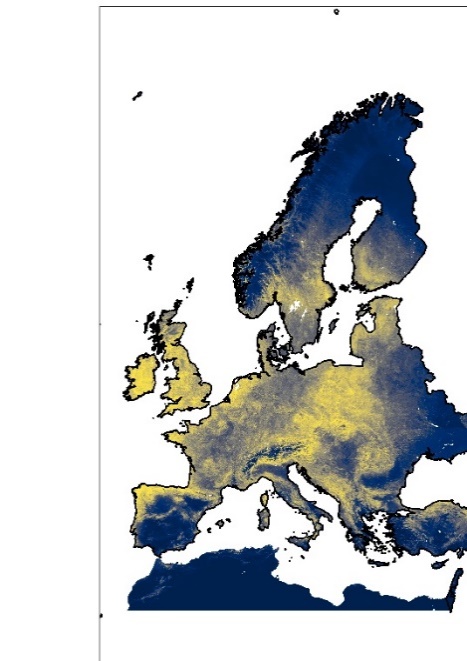


*Plecotus auritus*


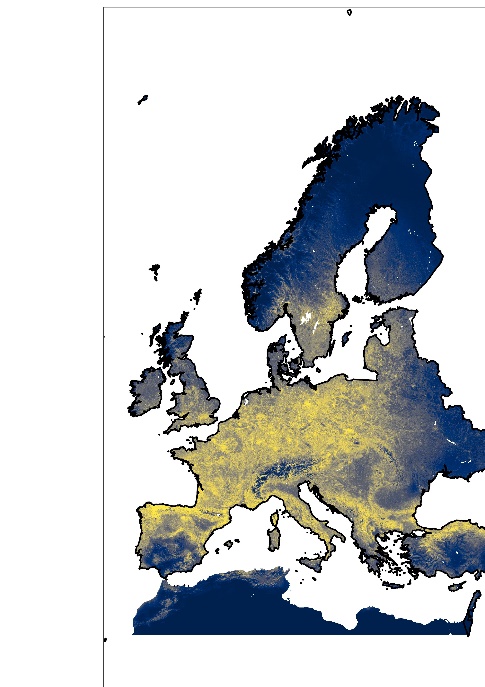


*Plecotus austriacus*


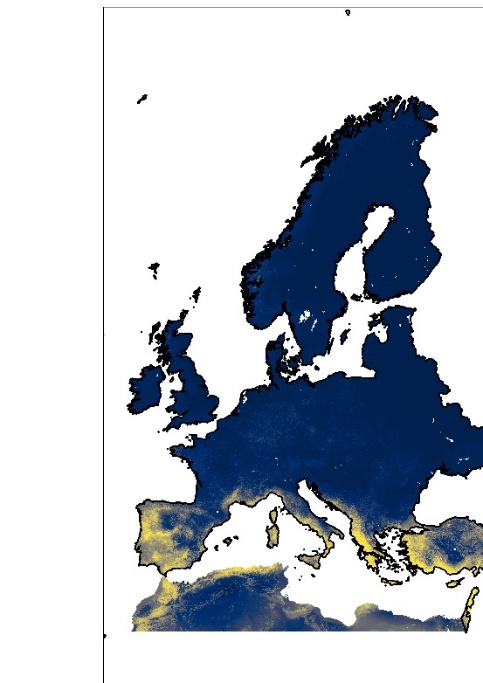


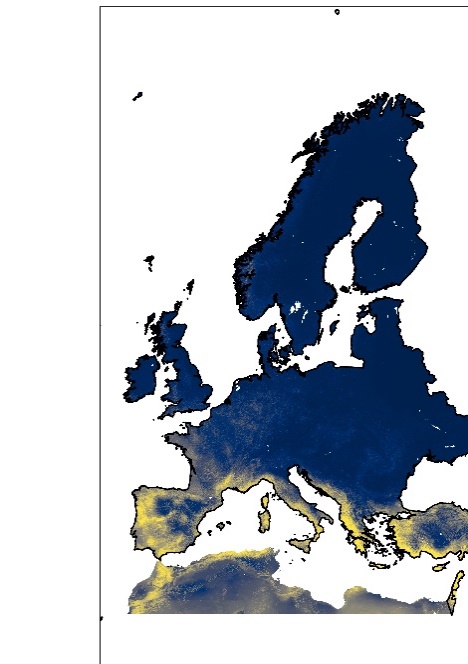

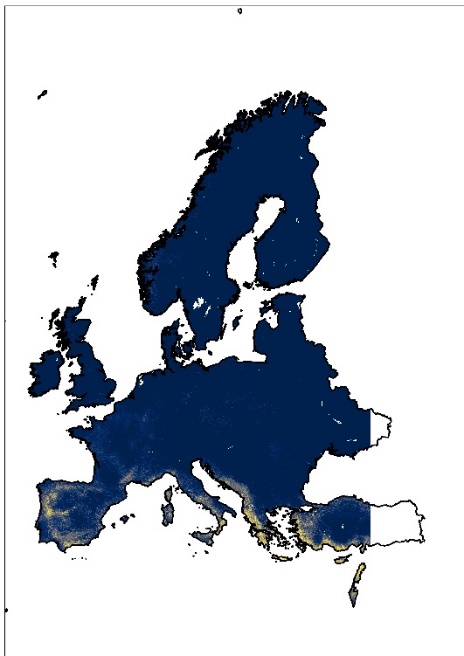


*Plecotus kolombatovici*


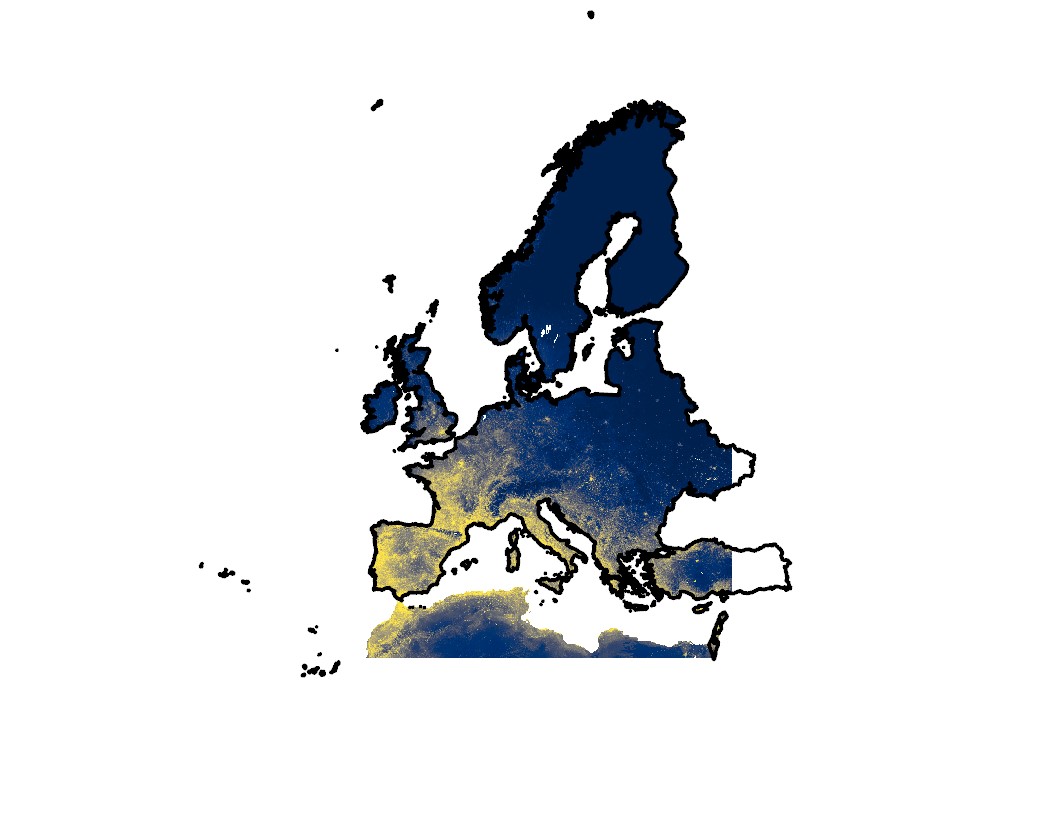

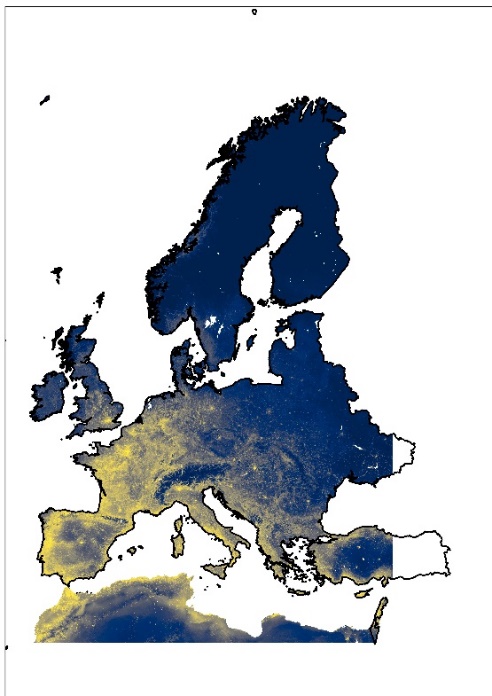

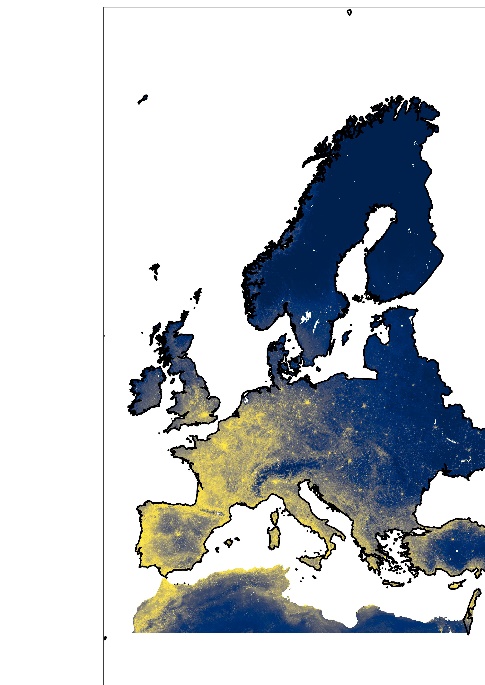


*Pipistrellus kuhli*

*Plecotus macrobullaris*

*Pipistrellus nathusii*

*Pipistrellus pipistrellus*

*Pipistrellus pygmaeus*

*Rhinolophus blasii*

*Rhinolophus euryale*

*Rhinolophus ferrumequinum*

*Rhinolophus hipposideros*

*Rhinolophus mehelyi*

*Tadarida teniotis*

*Vespertilio murinus*

**Appendix S9**. Predicted range suitability for 37 European bat species under current and future conditions (RCP 4.5 and RCP 8.5 emission scenarios) based on ensemble modeling.

**Appendix S10.** Random forest variable importance estimates indicating the relative contribution of the 37 bat species to the change in functional diversity.
